# Significance of halogen bonding in the synergistic nucleation of iodine oxoacids and iodine oxides†

**DOI:** 10.1039/d5sc02517f

**Published:** 2025-07-21

**Authors:** Rongjie Zhang, Yueyang Liu, Rujing Yin, Fangfang Ma, Deming Xia, Jingwen Chen, Hong-Bin Xie, Joseph S. Francisco

**Affiliations:** a Key Laboratory of Industrial Ecology and Environmental Engineering (Ministry of Education), School of Environmental Science and Technology, Dalian University of Technology Dalian 116024 China hbxie@dlut.edu.cn; b Department of Earth and Environmental Science, University of Pennsylvania Philadelphia PA 19104-6316 USA frjoseph@sas.upenn.edu

## Abstract

Congeneric iodine oxoacids and iodine oxides, key nucleating vapours in the marine atmosphere, have been reported to nucleate individually. However, whether they can nucleate together remains unknown. Here, we provide molecular-level evidence that I_2_O_4_, the iodine oxide with the highest nucleation potential towards iodine oxoacids, can synergistically nucleate with HIO_3_–HIO_2_. The nucleation rate of HIO_3_–HIO_2_–I_2_O_4_ is 1.5 to 6.8 times higher than that of the known most efficient iodine-associated two-component (HIO_3_–HIO_2_) nucleation at 278.15 K, enhancing the role of iodine-containing species in marine atmospheric particle formation. Microscopic analysis of the three-component cluster configurations revealed that an unexpected acid–base reaction between I_2_O_4_ and HIO_2_/HIO_3_ is a key driver of this efficient synergistic nucleation, besides hydrogen bonds and halogen bonds. We identified halogen bond-induced basicity enhancement as the chemical nature of I_2_O_4_ behaving as a base in the nucleation process with HIO_2_/HIO_3_. Such a basicity enhancement effect can be extended to other iodine-containing species, *e.g.*, HIO_2_ and the more acidic HIO_3_, suggesting a common feature in interactions between iodine-containing species. Our findings clarify the synergistic nucleation of iodine oxoacids and iodine oxides and highlight the necessity of considering the effect of halogen bond-induced basicity enhancement on the formation of iodine-containing particles.

## Abstract 2

I_2_O_4_ was found to synergistically nucleate with HIO_3_–HIO_2_ and halogen bond-induced basicity enhancement was identified as the chemical nature of I_2_O_4_ behaving as a base in the nucleation with HIO_3_/HIO_2_.

## Graphical abstract

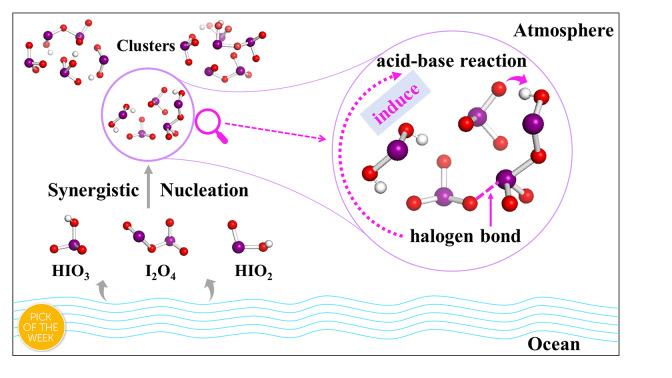

## Introduction

New particle formation (NPF) initiated by the nucleation of condensable vapours produces more than half of all atmospheric aerosols.^1–3^ These nascent particles act as cloud condensation nuclei (CCN), significantly influencing the earth's atmosphere radiative balance and modulating global climate patterns.^4,5^ Marine NPF plays a pivotal role in the global climate model, as marine clouds have high albedo and are susceptible to changes in CCN availability.^6–10^ However, the influence of marine NPF on climate remains uncertain, mainly due to the limited understanding of the nucleation process in the marine atmosphere, thus resulting in extensive uncertainty within global models.

Conventional theory has been based on the assumption that sulfuric acid (SA)–ammonia (NH_3_) nucleation is the dominant mechanism in NPF in marine environments.^11,12^ However, recent research has highlighted the importance of iodine oxides (I_*x*_O_*y*_) and iodine oxoacids (HIO_*z*_) as crucial nucleation precursors.^10,13–28^ These species are formed mainly by the transformation of iodine (I_2_) and methyl iodide (CH_3_I), which are emitted from the ocean surface.^29–33^ Although iodine-initiated NPF events have been frequently observed,^13,34–37^ the specific nucleation mechanism is still not fully understood. One study reported that in flow tube experiments, the nucleation of iodine oxides, particularly I_2_O_2_, I_2_O_3_, and I_2_O_4_ at high concentrations, is rapid.^14^ However, Cosmics Leaving Outdoor Droplets (CLOUD) chamber experiments have shown that nucleation involving iodic acid (HIO_3_) and iodous acid (HIO_2_) can occur rapidly under marine boundary layer conditions.^38^ In these CLOUD chamber experiments, the concentration of formed iodine oxides is relatively low when the precursor concentration is low, which could be one of the reasons why iodine oxide nucleation cannot compete with HIO_3_–HIO_2_ nucleation. Iodine oxides and iodine oxoacids are known to coexist on the basis of their homology and can form strong halogen bonds (typical halogen bond energies Δ*E* ranging from 1 to 45 kcal mol^−1^ calculated by Oliveira *et al.*^39^), which are non-covalent interactions formed between an electrophilic halogen atom and a nucleophilic heteroatom (*i.e.*, with lone-pair electrons).^39,40^ Therefore, they may be able to nucleate together, so investigating the joint nucleation mechanism for iodine oxides and iodine oxoacids is important.

In this study, the multicomponent nucleation mechanism of iodine oxides and iodine oxoacids was investigated *via* quantum chemical calculations and atmospheric cluster dynamic coding. Here, multicomponent nucleation was considered to occur among HIO_3_, HIO_2_ and specific iodine oxides since HIO_3_ and HIO_2_ have been found to be able to nucleate at realistic atmospheric concentration ranges.^38,41^ Since dimer can present the foundational intermolecular interaction between iodine oxides and iodine oxoacids, and the computational cost of dimer is low, the dimer formation free energy (Δ*G*) between iodine oxides (IO, I_2_O_2_, I_2_O_3_, I_2_O_4_, and I_2_O_5_) and iodine oxoacids (HIO_3_ and HIO_2_) was used to screen the propensity of specific iodine oxides towards nucleation with iodine oxoacids. I_2_O_4_ was found to have the greatest nucleation potential among the selected iodine oxides and can efficiently nucleate with HIO_3_–HIO_2_ as a base. Halogen bond-induced basicity enhancement of I_2_O_4_ was found to be the chemical nature for I_2_O_4_ behaving as a base during the nucleation with HIO_3_–HIO_2_. This study reveals that iodine oxides and iodine oxoacids can synergistically nucleate, breaking through the previous findings of their independent nucleation^14,38^ and providing a new chemical mechanism for iodine-containing particles.

## Methods

A multistep sampling scheme was employed to identify the global minimum for all of the studied clusters, including I_*x*_O_*y*_–HIO_2–3_ dimer clusters and HIO_3_–HIO_2_–I_2_O_4_ system clusters. This procedure has been widely used in many previous studies on atmospheric cluster formation.^42–44^ First, 5000–8000 initial configurations were generated for each cluster using the ABCluster program^45^ and were optimized *via* the semiempirical PM7 method. The single point energy was subsequently calculated at the M06-2X/def2-TZVP level for all the converged geometries. Configurations up to 15 kcal mol^−1^ higher than the lowest energy configuration were further fully optimized at the M06-2X/6-31++G(d,p) (for H O atoms) + aug-cc-pVTZ-PP (a basis set with relativistic pseudopotential for I atoms^46^) level. Since there are tens of configurations we have to optimize for each cluster in this step, the adopted larger basis set such as aug-cc-pVTZ(-PP) (aug-cc-pVTZ for H O atoms and aug-cc-pVTZ-PP for I atoms) would lead to the high-cost for the computational time. Hence, we employed the smaller basis set here to reduce computational cost. Our previous study found that the adopted basis set here for optimization presents similar Δ*G* for HIO_3_–HIO_2_ clusters to that of the aug-cc-pVTZ(-PP) basis set (Table S1†).^41^ Moreover, we also tested several clusters including (HIO_2_)_1_(I_2_O_4_)_1_, (HIO_3_)_2_(I_2_O_4_)_2_ and (HIO_3_)_1_(HIO_2_)_1_(I_2_O_4_)_1_ with the other basis set def2-TZVP. It was found that the low-energy configurations (within 1–2 kcal mol^−1^ of the lowest-energy configuration) from def2-TZVP basis set are the same as those from 6-31++G(d,p) + aug-cc-pVTZ-PP (Fig. S1†). Therefore, the adopted basis set is acceptable for the first-round geometry optimization for HIO_3_–HIO_2_–I_2_O_4_ system. Finally, for the lowest-energy configurations within 1–2 kcal mol^−1^, reoptimization was performed at the M06-2X/aug-cc-pVTZ(-PP) level, and the single point energy was refined at the DLPNO-CCSD(T)/aug-cc-pVTZ(-PP) level. If the calculation results failed during optimization or ended up with imaginary frequencies, the initial configurations were adjusted and reoptimized until a successful optimization without imaginary frequencies was finished. The configuration with the lowest Gibbs free energy (*G*) at 298.15 K and 1 atm was chosen as the global minimum. The *G* values at other temperatures and pressures were obtained *via* a GoodVibes Python script.^47^ The Δ*G* for the global minimum was calculated by subtracting the *G* of the constituent molecules from that of the cluster. We also tested the influence of the dispersion correction by reoptimizing the global minima for selected three clusters ((HIO_2_)_1_(I_2_O_4_)_1_, (HIO_3_)_2_(I_2_O_4_)_2_ and (HIO_3_)_1_(HIO_2_)_1_(I_2_O_4_)_1_) using M06-2X/aug-cc-pVTZ(-PP), with Grimme's dispersion correction with ZERO Damping GD3.^48–50^ The root-mean-square deviation (RMSD) for optimized structure and difference in Δ*G* from method with and without dispersion correction are 0.001–0.003 Å and −0.004–0.012 kcal mol^−1^, indicating that the dispersion correction causes slight influence on the results of this study (Table S2†). All the PM7 and M06-2X calculations were carried out with the Gaussian 16 program^51^ and DLPNO-CCSD(T) calculations were performed with the software ORCA 5.0.3.^52,53^ In addition, natural bond orbital (NBO) analysis was carried out by Gaussian 16 program to give a detailed insight into intermolecular interactions.

The cluster formation rate and growth mechanism were analysed *via* Atmospheric Cluster Dynamics Code (ACDC) software, which simulates the cluster kinetics by means of explicit solution of the birth–death equations.^54^ Herein, the simulated system was treated as a 3 × 3 box containing (HIO_3_)_0–3_(HIO_2_)_0–3_, (HIO_3_)_0–3_(I_2_O_4_)_0–3_, (HIO_2_)_0–3_(I_2_O_4_)_0–3_ and (HIO_3_)_*x*_(HIO_2_)_*y*_(I_2_O_4_)_*z*_ (*x* = 1–3, *y* + *z* = 2–3) clusters. The boundary cluster settings can be found in the ESI.† The simulation mainly ran at 278.15 K and 1 atm with a coagulation sink rate coefficient (*k*_coag_) of 2 × 10^−3^ s^−1^, a typical value in coastal regions where iodine species are nucleating.^55^ Additional simulations were performed at 298.15, 253, 223.15 K to probe the effect of temperature, and further simulations were conducted at 0.5 and 0.1 atm to test the effect of pressure. The concentration of HIO_3_ ([HIO_3_]) was set to 10^5^ to 10^8^ cm^−3^ based on the common atmospheric concentration range.^13,36–38^ The concentrations of HIO_2_ and I_2_O_4_ were set to approximately 1/30 [HIO_3_] and 1/100 [HIO_3_], respectively, corresponding to the measured steady-state concentrations in the CLOUD chamber experiment.^38^ The detailed discussion on the selection of [I_2_O_4_] was presented in the ESI.† We accounted for the contribution of long-range interactions *via* an enhancement factor of 2.4 for the collision rate coefficient.^41,56^ In addition, the cluster formation rate of the SA–HIO_3_–HIO_2_ system ((SA)_*x*_(HIO_3_)_*y*_(HIO_2_)_*z*_ (0 ≤ *x* + *y* ≤ 3, 1 ≤ *z* ≤ 3) clusters) was simulated for comparison, with the concentration of SA ranging from 10^6^ to 10^7^ cm^−3^.^37,57^

The similar ACDC simulations based on thermodynamics data from the same theoretical methods as ones used here have been employed in the previous studies on SA–HIO_3_–HIO_2_ and HIO_3_–HIO_2_ nucleation, producing consistent cluster formation rates with those from CLOUD experiments.^26,41^ Therefore, it is reasonably believed that adopted computational level of theories combined with ACDC simulations can be reliable to describe the interactions among the iodine species and predict the cluster formation mechanism for the HIO_3_–HIO_2_–I_2_O_4_ system.

To investigate the correlation between the formation of halogen bonds (XBs) and the change in basicity of I_2_O_4_, a series of I_2_O_4_-containing dimer clusters that contain only one XB were manually constructed and optimized at the M06-2X/aug-cc-pVTZ(-PP) level. After optimization, the single point energies (*E*) of these dimer clusters were calculated at the DLPNO-CCSD(T)/aug-cc-pVTZ(-PP) level. The bond energy (Δ*E*) was calculated to represent the strength of the formed XB:

1 Δ*E* = |*E*(precursor–I_2_O_4_ dimer) − *E*(precursor) − *E*(I_2_O_4_)|

Previous study has shown that the change in electrostatic potential (ΔESP) at specific basic site can be employed to present the change in basicity of the compounds.^58^ Herein, we employed ΔESP for the basic site of I_2_O_4_ to represent the change in basicity of I_2_O_4_ when it forms XBs with various precursors. The computational details for ΔESP was presented in the ESI.† Furthermore, we also directly calculated the gas basicity (GB) of I_2_O_4_ before and after the formation of XBs, which is defined as the Gibbs free energy change (Δ*G*) for reactions (2) and (3), respectively:

2 I_2_O_4_H^+^ (g) → I_2_O_4_ (g) + H^+^ (g)

3 precursor–I_2_O_4_H^+^ (g) → precursor–I_2_O_4_ (g) + H^+^ (g)

It is noted that the terminal O atom of I_2_O_4_ with the most negative average ESP after forming XB was taken as the basic site for ΔESP and GB calculation. The geometries of positive precursor–I_2_O_4_H^+^ dimers and I_2_O_4_H^+^ were manually constructed by adding H^+^ to the basic site of I_2_O_4_.

## Results and discussion

### Of all iodine oxides, I_2_O_4_ is the precursor with the highest potential towards iodine oxoacids

**Fig. 1:**
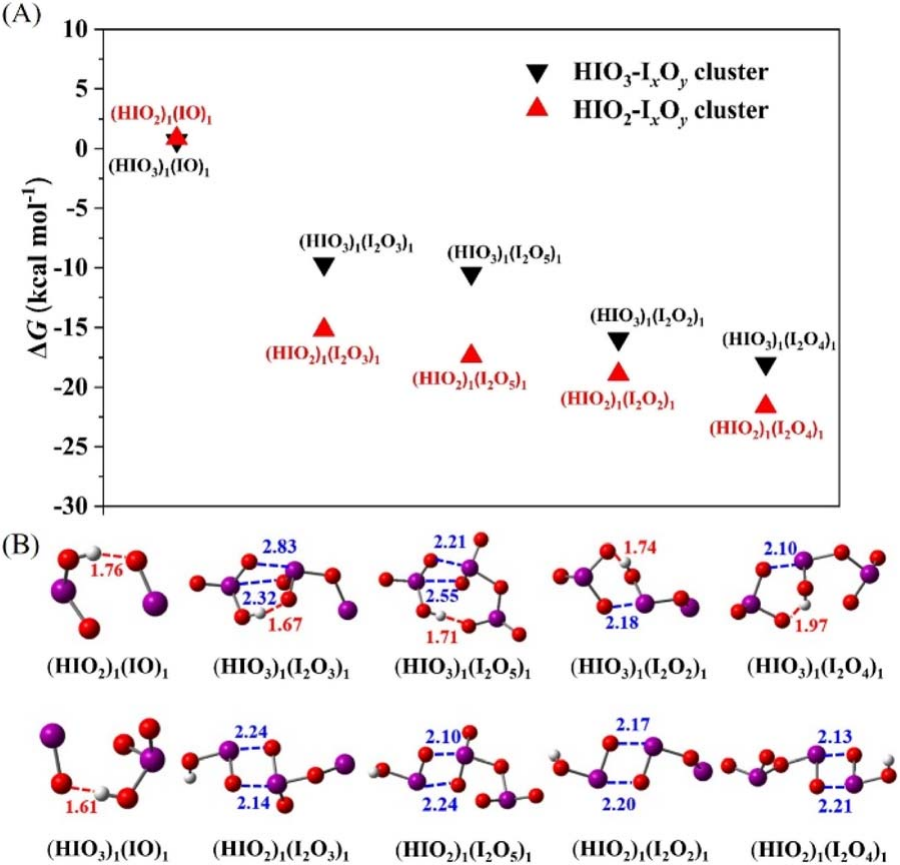
Formation free energy (Δ*G*) (A) and global minimum configurations (B) of I_*x*_O_*y*_–HIO_2–3_ dimers at the DLPNO-CCSD(T)/aug-cc-pVTZ(-PP)//M06-2X/aug-cc-pVTZ(-PP) level of theory at 298.15 K. The red balls represent oxygen atoms, purple ones are for iodine atoms, and white ones are for hydrogen atoms. The red dashed lines in the configurations represent hydrogen bonds (HBs), and the blue dashed lines represent XBs. The red and blue numbers indicate the bond lengths of HBs and XBs, respectively. The bond lengths are given in Å.

The nucleation potential of iodine oxides towards iodine oxoacids was checked by their dimerization with HIO_2_ or HIO_3_. Fig. 1 presents the Δ*G* values and global minimum configurations of the HIO_2_-containing and HIO_3_-containing dimer clusters. The Δ*G* values for all dimers except IO–HIO_2_ and IO–HIO_3_ are negative, ranging from −9.7 to −21.6 kcal mol^−1^. Of all the HIO_2_-containing and HIO_3_-containing dimers, HIO_2_–I_2_O_4_ and HIO_3_–I_2_O_4_ exhibit the lowest Δ*G* values, followed by HIO_2_–I_2_O_2_ and HIO_3_–I_2_O_2_, respectively. Therefore, I_2_O_4_ is expected to have the greatest intrinsic nucleation potential towards iodine oxoacids, followed by I_2_O_2_. In addition, the Δ*G* values of HIO_2_–I_2_O_4_ and HIO_3_–I_2_O_4_ are even lower than that of HIO_3_–HIO_2_ (−16.79 kcal mol^−1^), indicating that I_2_O_4_ has a greater ability to bind with HIO_2_ or HIO_3_ than HIO_3_ and HIO_2_ do with each other. It is noteworthy that Engsvang *et al.* also calculated Δ*G* values for dimers of iodine oxides (I_2_O_4_ and I_2_O_5_) with iodine oxoacids in a recent study.^59^ They found I_2_O_4_–HIO_2–3_ dimers have lower Δ*G* values than I_2_O_5_–HIO_2–3_ dimers, consistent with results predicted here, although the specific Δ*G* values in this study are lower.

Observation of the configurations and NBO analysis of the dimers found that both I_2_O_4_ and I_2_O_2_ accept a proton from HIO_3_ in HIO_3_–I_2_O_4_ and HIO_3_–I_2_O_2_ clusters in addition to forming XBs and hydrogen bonds (HBs). Accordingly, both I_2_O_4_ and I_2_O_2_ behave as bases towards HIO_3_. However, for dimers of other iodine oxides with HIO_3_, there is no proton transfer even though XBs and HBs are formed. Therefore, the additional electrostatic interaction caused by the proton transfer reaction is expected to be one reason that I_2_O_4_ and I_2_O_2_ have lower dimer Δ*G* values with HIO_3_ than other iodine oxides do. It is noted that the structure of (HIO_3_)_1_(I_2_O_4_)_1_ resembles two IO_3_ radicals clustered with a hypoiodous acid HOI. However, (HIO_3_)_1_(I_2_O_4_)_1_ could not dissociate into HOI + 2·IO_3_ rather than HIO_3_ + I_2_O_4_, since the Δ*G* value for (HIO_3_)_1_(I_2_O_4_)_1_ dissociating into HOI + 2·IO_3_ (76.14 kcal mol^−1^) is much higher than HIO_3_ + I_2_O_4_ (18.01 kcal mol^−1^). For the dimers of HIO_2_ with I_2_O_4_ and I_2_O_2_, two XBs are formed. Two XBs are known to form for the dimers of HIO_2_ with other iodine oxides except for IO. The lower dimer Δ*G* values of I_2_O_4_ and I_2_O_2_ with HIO_2_ result from the stronger strength of XBs formed between them, as evidenced by the lower average energy gap between antibonding orbital δ*(O–I) and lone-pair orbital LP(O), which are two critical molecular orbitals for the formation of XBs with HIO_2_ (Table S3†). As a representative example, Fig. S2† illustrates the δ*(O–I) and LP(O) orbitals involved in the (I_2_O_4_)_1_(HIO_2_)_1_ dimer. Therefore, although I_2_O_5_ has more electronically deficient iodine centers than I_2_O_4_/I_2_O_2_, the additional electrostatic interaction caused by the proton transfer reaction between I_2_O_4_/I_2_O_2_ and HIO_3_, and the stronger average strength of two XBs between I_2_O_4_/I_2_O_2_ and HIO_2_, make I_2_O_4_/I_2_O_2_ form a more stable complex with iodine oxoacids compared to I_2_O_5_.

As discussed above, I_2_O_4_ is expected to have the highest intrinsic nucleation potential towards iodine oxoacids. In addition, in photochemical reactions of I_2_ or CH_3_I with ozone (O_3_) under laboratory conditions, the gaseous concentration of I_2_O_4_ produced was higher than that of all other iodine oxides except IO.^15,60^ Therefore, I_2_O_4_ has the greatest nucleation potential towards iodine oxoacids. In the following section, we describe our detailed investigation of the joint nucleation of I_2_O_4_ and iodine oxoacids.

### Configurations of HIO_3_–HIO_2_–I_2_O_4_ clusters featuring halogen-induced acid–base reaction

**Fig. 2:**
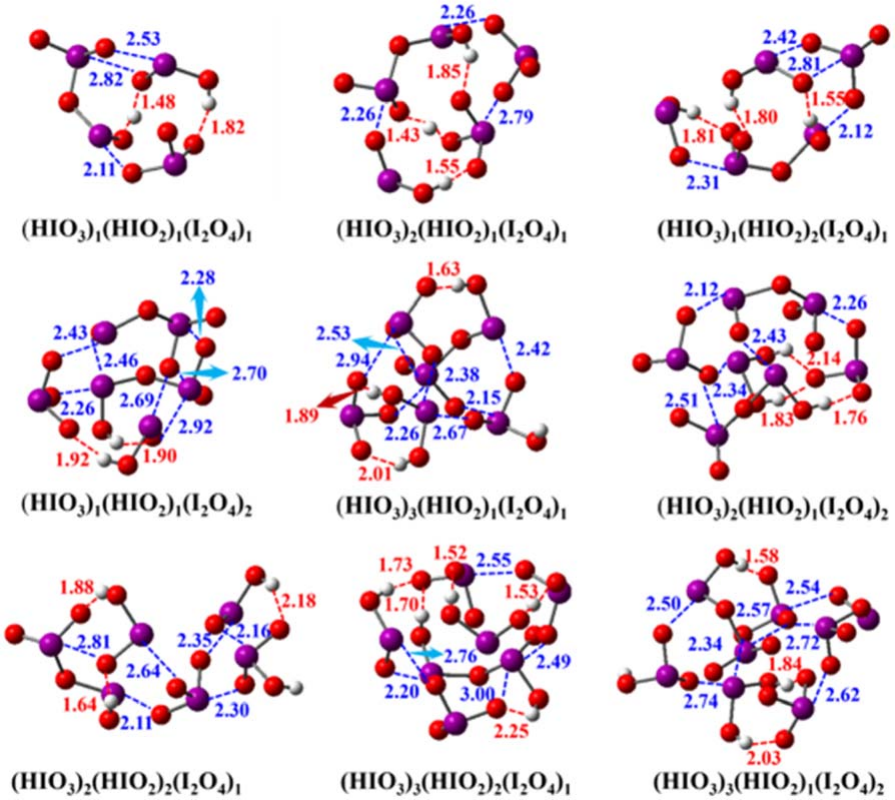
Global minimum configurations of the (HIO_3_)_*x*_(HIO_2_)_*y*_(I_2_O_4_)_*z*_ (*x* = 1–3, *y* + *z* = 2–3) clusters calculated at the DLPNO-CCSD(T)/aug-cc-pVTZ(-PP)//M06-2X/aug-cc-pVTZ(-PP) level of theory. The red balls represent oxygen atoms, purple ones are for iodine atoms, and white ones are for hydrogen atoms. The red dashed lines represent HBs, and the blue dashed lines represent XBs. The red and blue numbers indicate the bond lengths of HBs and XBs, respectively. The bond lengths are given in Å.

We present the cluster configurations for the HIO_3_–HIO_2_–I_2_O_4_ system in Fig. 2 and S3.† Since HIO_3_–HIO_2_ configurations have already been investigated in our previous study,^41^ we mainly focus on HIO_3_–I_2_O_4_ and HIO_2_–I_2_O_4_ two-component clusters (Fig. S3†) and HIO_3_–HIO_2_–I_2_O_4_ three-component clusters (Fig. 2) here. Observation and analysis of the cluster configurations shows that I_2_O_4_ accepts a proton from HIO_3_ and HIO_2_ in almost all HIO_3_–I_2_O_4_ and HIO_2_–I_2_O_4_ two-component clusters. Therefore, I_2_O_4_ behaves as a Brønsted–Lowry base when it clusters with HIO_3_ or HIO_2_. To our knowledge, this is the first time that I_2_O_4_ has been revealed to behave as a base in clustering with HIO_3_ or HIO_2_. In HIO_3_–HIO_2_–I_2_O_4_ three-component clusters, only HIO_3_, with the greater acidity, can donate a proton, and I_2_O_4_ or HIO_2_ can accept a proton. In addition, I_2_O_4_ has a greater ability to accept a proton from HIO_3_ than HIO_2_ does. Therefore, the behaviour of I_2_O_4_ as a base is a common feature of HIO_3_–I_2_O_4_ and HIO_2_–I_2_O_4_ two-component clusters and HIO_3_–HIO_2_–I_2_O_4_ three-component clusters, in addition to the formation of typical XBs and HBs.

Why I_2_O_4_ can behave as a base towards HIO_2–3_ is an interesting topic for discussion. The calculated gas basicity (GB) of I_2_O_4_ is 199 kcal mol^−1^, close to that of NH_3_ (196 kcal mol^−1^), which has been found to be unable to accept a proton from HIO_3_ in the (HIO_3_)_1_(NH_3_)_1_ dimer.^17,18^ Therefore, the GB of I_2_O_4_ cannot explain its acid–base reaction with HIO_2_ and HIO_3_. According to observation of the configurations of HIO_3_–HIO_2_–I_2_O_4_ clusters, the occurrence of acid–base reactions with I_2_O_4_ as a base is always accompanied by the formation of XBs. Therefore, it is reasonable to speculate that the formation of XBs can induce the basicity enhancement of I_2_O_4_, further driving the acid–base reaction.

**Fig. 3:**
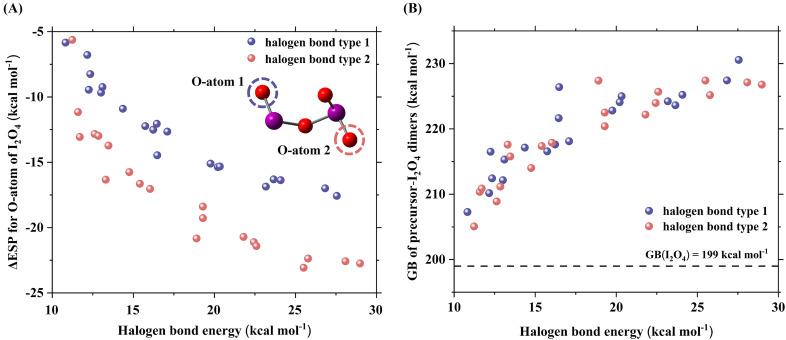
Change in electrostatic potential (ΔESP) values (kcal mol^−1^) for the O atom as a basic site of I_2_O_4_ (A) and the gas basicity (GB) (kcal mol^−1^) of precursor–I_2_O_4_ dimers (B) as a function of the halogen bond energy (kcal mol^−1^). The purple and red spheres represent two different types of XBs. O-atom 1 is the basic site of I_2_O_4_ when it forms XB type 1 with precursors. O-atom 2 is the basic site of I_2_O_4_ when it forms XB type 2 with precursors.

To verify this speculation, ΔESP for the basic site and GB of I_2_O_4_ when it forms XBs with various precursors, were determined. Here, the selected precursors included amines, carbonyl compounds, sulfides, alcohols, benzothiazole, peroxides and hydroperoxymethyl, which can form XBs with I_2_O_4_ at various bond strengths. Since I_2_O_4_ can form two types of XBs *via* its two lowest energy antibonding orbitals δ*(O–I), the effects of these two types of XBs on ΔESP and GB were considered. Fig. 3A shows that the ΔESP values for the O atom as a basic site are negative when I_2_O_4_ forms a XB with precursors (identity of selected precursors was presented in Table S4†), which leads to an increased basicity of I_2_O_4_. Moreover, a higher XB energy generally results in a greater (more negative) ΔESP value. As shown in Fig. 3B, the GB of I_2_O_4_–precursor dimer clusters is significantly greater than that of the I_2_O_4_ monomer regardless of the type of XBs. In addition, a roughly positive correlation between the formed XB energy and the GB values of I_2_O_4_–precursor dimer clusters is observed. More importantly, even when a relatively weak XB (approximately 13 kcal mol^−1^) is formed, the GB of I_2_O_4_ can be increased to 215 kcal mol^−1^, which is close to that of dimethylamine (DMA) (215 kcal mol^−1^), a typical base for enhancing SA-driven nucleation.^57^ Therefore, the formation of XBs indeed increases the basicity of I_2_O_4_, increasing it from a level similar to that of NH_3_ to a level that is comparable to or even greater than that of DMA.

Notably, the present study and an earlier study also revealed that HIO_2_ behaves as a base towards HIO_3_.^41^ Similarly, the ΔESP for the basic site of HIO_2_ when it forms XBs with various precursors was determined. An effect similar to that of the formed XB on the ΔESP for the terminal O atom as a basic site was also found for the HIO_2_ system (Fig. S4†). Therefore, the formation of XBs can greatly increase the basicity of I_2_O_4_ and HIO_2_. To our knowledge, this is the first study to reveal that XBs induce acid–base reactions in HIO_3_–HIO_2_ and HIO_3_–HIO_2_–I_2_O_4_ nucleation systems.

Since XBs are weak non-covalent interactions, it is interesting to check whether other weak non-covalent interactions like HBs, commonly formed in the acid–base nucleation system, can also induce the basicity enhancement of compounds. Here, possible basicity enhancement effect of the HBs was checked by taking NH_3_ as the test case. It was found that HBs indeed can induce the basicity enhancement of NH_3_ (see ΔESP values for the N atom as the basic site of NH_3_ when it forms HBs with precursors in Table S5†). Still, when XBs and HBs are considered as non-covalent interactions, the correlation of non-covalent interaction energy with ΔESP is similar to that of XB energy with ΔESP, *i.e.* a higher non-covalent interaction energy generally results in a greater (more negative) ΔESP value (Fig. S5†). Since the HB energy is weaker than XB energy considered here, the basicity enhancement effect of HBs is much lower than that of the XBs (Fig. S5†). If the stronger or more HBs are formed in the SA–base systems, the basicity enhancement effect of HBs could be improved. In view of potential role of HBs in inducing basicity enhancement, future investigation on effect of HB-induced basicity enhancement in different nucleation systems is warranted.

### Low formation free energy of I_2_O_4_-containing clusters

The cluster formation free energy of the HIO_3_–HIO_2_–I_2_O_4_ system at 278.15 K and 1 atm is presented in Fig. S6.† As shown in Fig. S6,† the Δ*G* values of all HIO_3_–I_2_O_4_ and HIO_2_–I_2_O_4_ two-component clusters are lower than those of the corresponding HIO_3_–HIO_2_ two-component clusters. The Δ*G* values of all I_2_O_4_-rich clusters are lower than those of the corresponding HIO_3_ or HIO_2_-rich clusters in these two-component clusters. In addition, the Δ*G* values of the I_2_O_4_-containing three-component clusters are lower than those of the corresponding HIO_3_–HIO_2_ two-component clusters with the same number of acids (HIO_3_) and bases (HIO_2_ and I_2_O_4_). All of the aforementioned results indicate that I_2_O_4_ has a greater ability to bind with HIO_2_ or HIO_3_ than HIO_3_ and HIO_2_ do with each other, which is consistent with the conclusion from the Δ*G* analysis of the dimers of I_2_O_4_ with HIO_2_ and HIO_3_. The higher binding ability of I_2_O_4_ leads to the high stability of most I_2_O_4_-containing clusters, as evidenced by their low evaporation rates (Fig. S7†). Therefore, the participation of I_2_O_4_ in HIO_3_–HIO_2_ nucleation may be favorable. It is noteworthy that (HIO_2_)_3_(I_2_O_4_)_3_ and (HIO_2_)_3_(I_2_O_4_)_2_ clusters own high evaporation rates despite they have low Δ*G* values. It is understandable since the evaporation rate is not only dependent on the Δ*G* value of the individual cluster, but also on the stability of its all possible daughter clusters.^54^ The high evaporation rate of (HIO_2_)_3_(I_2_O_4_)_3_ and (HIO_2_)_3_(I_2_O_4_)_2_ clusters result from the higher stability of their daughter clusters (*e.g.* (HIO_2_)_2_(I_2_O_4_)_2_). Tables S6 and S7† also display the Δ*G* values of the HIO_3_–HIO_2_–I_2_O_4_ system clusters at other temperatures and pressures. The Δ*G* values of most clusters become more negative with decreasing temperature, while they become higher with decreasing pressure.

Previous experimental and theoretical studies have shown that HBs are favorable for new particle formation, particularly for those involving organic acids.^61,62^ Herein, we also compared the effects of HBs and XBs on cluster formation by examining the formation free energy of two types of specific dimers: organic acids (benzoic acid, *cis*-pinonic acid, formic acid)–NH_3_ dimers formed by HBs,^62^ and HIO_2_–I_2_O_4_ and HIO_2_–HIO_2_ dimers formed by XBs.^41^ It is found that the formation free energies of HIO_2_–I_2_O_4_ and HIO_2_–HIO_2_ dimers are lower than those of organic acids (benzoic acid, *cis*-pinonic acid, formic acid)–NH_3_ dimers (Table S8†). This implies that XBs here are stronger than HBs.

### Cluster formation rates of the HIO_3_–HIO_2_–I_2_O_4_ system

**Fig. 4:**
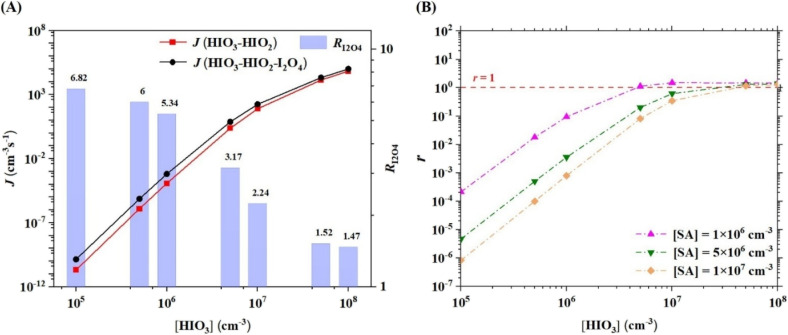
Cluster formation rates (*J*) (cm^−3^ s^−1^) of the HIO_3_–HIO_2_ and HIO_3_–HIO_2_–I_2_O_4_ systems and enhancement coefficient (*R*_I_2_O_4__) (the ratio of the *J* value of the HIO_3_–HIO_2_–I_2_O_4_ system relative to that of the HIO_3_–HIO_2_ system) (A) and comparison coefficient (*r*) (the ratio of the *J* value of the HIO_3_–HIO_2_–I_2_O_4_ system relative to that of the SA–HIO_3_–HIO_2_ system) (B) as a function of precursor concentration at 278.15 K, 1 atm and *k*_coag_ = 0.002 s^−1^.

The variation in the cluster formation rates (*J*) of the three-component HIO_3_–HIO_2_–I_2_O_4_ system with [HIO_3_] (10^5^–10^8^ cm^−3^) ([HIO_2_] = 1/30 [HIO_3_], I_2_O_4_ = 1/100 [HIO_3_]) at 278.15 K, 1 atm and *k*_coag_ = 0.002 s^−1^, along with that of the two-component HIO_3_–HIO_2_ system, is presented in Fig. 4A. The detailed computational methods for *J* are presented in ESI.† Note that pure I_2_O_4_ nucleation is not compared with three-component nucleation since its rate is much lower than that of HIO_3_–HIO_2_ nucleation when [I_2_O_4_] = 1/100 [HIO_3_] (Fig. S8†). As shown in Fig. 4A, the *J* values of the HIO_3_–HIO_2_–I_2_O_4_ system increase with [HIO_3_]. The *J* values of the HIO_3_–HIO_2_–I_2_O_4_ system are higher than those of the two-component HIO_3_–HIO_2_ system under the studied conditions, especially at low [HIO_3_]. Therefore, I_2_O_4_ can enhance HIO_3_–HIO_2_ nucleation. To clearly demonstrate the enhancement effect of I_2_O_4_, the enhancement coefficient (*R*_I_2_O_4__), which is the ratio of the *J* value of the HIO_3_–HIO_2_–I_2_O_4_ system relative to that of the HIO_3_–HIO_2_ system, was calculated. The calculated *R*_I_2_O_4__ ranges from 1.5 to 6.8 at 278.15 K, depending on [HIO_3_]. The lower [HIO_3_] is, the higher *R*_I_2_O_4__ is. The *R*_I_2_O_4__ of 6.8 applies when the absolute *J* is very low and the maximum *R*_I_2_O_4__ for non-negligible absolute *J* (around 7.5 cm^−3^ s^−1^) is about 2–3. The higher enhancement effect of I_2_O_4_ at low [HIO_3_] decreases the lower limit concentration that HIO_3_-driven nucleation has a pronounced nucleation rate. The required [HIO_3_] values are 3.45 × 10^6^ cm^−3^ and 4.35 × 10^6^ cm^−3^ for *J* values of approximately 1 cm^−3^ s^−1^ in the systems with and without I_2_O_4_, respectively. This provides further support for the idea that iodine oxides play a key role in iodine oxoacid nucleation. We also noted that a very recent study by Engsvang and Elm found “oxoacid-assisted oxide” nucleation efficient.^63^ In their study, it was found that the predicted rate of HIO_3_–HIO_2_ nucleation is much lower than the experimental rates based on their selected theoretical method.^63^ In our early studies, the predicted HIO_3_–HIO_2_ and SA–HIO_3_–HIO_2_ nucleation rates using the same theoretical methods as in this study are comparable to that from CLOUD data.^26,41^ Besides, the concentration of iodine oxide is much lower than that of iodine oxoacid in the ambient atmosphere.^38^ Hence, we treat HIO_3_–HIO_2_–I_2_O_4_ synergistic nucleation as “oxide-assisted oxoacid” here. In fact, both studies found that iodine oxoacid and iodine oxide can nucleate together.

The *J* of HIO_3_–HIO_2_–I_2_O_4_ system and *R*_I_2_O_4__ as a function of temperature at 1 atm are presented in Fig. S9.†*J* values rise as temperature declines, while further decreasing temperature exhibits a diminished effect on *J* below 253 K. The temperature dependence of *J* is more pronounced at lower [HIO_3_]. As shown in Fig. S9B,† enhancement of I_2_O_4_ on HIO_3_–HIO_2_ nucleation becomes higher at higher temperature (298.15 K). Fig. S10† illustrates the *J* and *R*_I_2_O_4__ as a function of pressure at 278.15 K. *J* of HIO_3_–HIO_2_–I_2_O_4_ system and *R*_I_2_O_4__ increase slightly as pressure decreases from 1 to 0.5 atm but remain nearly constant when pressure declines from 0.5 to 0.1 atm.

A recent study revealed that SA can also enhance HIO_3_–HIO_2_ nucleation.^26^ Therefore, comparing the enhancement potential of I_2_O_4_ with that of SA would be interesting. The variation in the comparison coefficient (*r*), which is the ratio of the *J* value of the HIO_3_–HIO_2_–I_2_O_4_ system relative to that of the SA–HIO_3_–HIO_2_ system, with [HIO_3_] and [SA], is presented in Fig. 4B. At low [HIO_3_] and therefore lower [I_2_O_4_], *r* is much less than 1, indicating that the enhancement potential of I_2_O_4_ is lower than that of SA. When [HIO_3_] ≥ 5 × 10^6^ cm^−3^ and the corresponding [I_2_O_4_] ≥ 5 × 10^4^ cm^−3^, *r* becomes approximately 1, *i.e.*, 1.12–1.50 for [SA] = 1 × 10^6^ cm^−3^; 0.20–1.38 for [SA] = 5 × 10^6^ cm^−3^, indicating that the enhancement potential of I_2_O_4_ is comparable to or even greater than that of SA for HIO_3_–HIO_2_ nucleation. In most cases for which *r* is approximately 1, [I_2_O_4_] is much lower than [SA]. Therefore, I_2_O_4_, as a base, has a greater enhancement efficiency than SA for HIO_3_–HIO_2_ nucleation.

### Synergistic nucleation mechanism of the HIO_3_–HIO_2_–I_2_O_4_ system

**Fig. 5:**
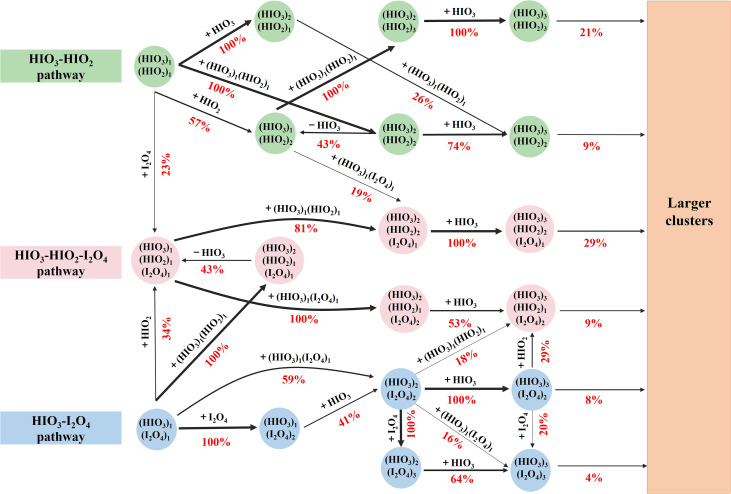
Cluster formation pathways for the HIO_3_–HIO_2_–I_2_O_4_ system at 278.15 K, [HIO_3_] = 1 × 10^7^ cm^−3^, [HIO_2_] = 3.33 × 10^5^ cm^−3^, [I_2_O_4_] = 1 × 10^5^ cm^−3^ and *k*_coag_ = 0.002 s^−1^. The pathways contributing less than 15% to the flux of cluster formation are not show.

The cluster growth pathway for the HIO_3_–HIO_2_–I_2_O_4_ system is shown in Fig. 5. Overall, the cluster growth pathway involves three channels: the (I) HIO_3_–HIO_2_ pathway, (II) HIO_3_–I_2_O_4_ pathway and (III) HIO_3_–HIO_2_–I_2_O_4_ pathway. The contributions of the two-component pathways (HIO_3_–HIO_2_ and HIO_3_–I_2_O_4_) are 30% and 12%, respectively, and the contribution of the HIO_3_–HIO_2_–I_2_O_4_ pathway is 38%. At the very early cluster formation stage, two-component clusters of HIO_3_–HIO_2_ and HIO_3_–I_2_O_4_ evolved into three-component HIO_3_–HIO_2_–I_2_O_4_ clusters. Note that almost all clusters growing out of the box had O/I ratios ranging from 2.3 to 2.5, which is consistent with the O/I ratio of approximately 2.5 reported in a previous laboratory study,^15^ supporting the occurrence of the proposed synergistic nucleation mechanism of iodine oxoacids and iodine oxides in the atmosphere.

## Implications

**Fig. 6:**
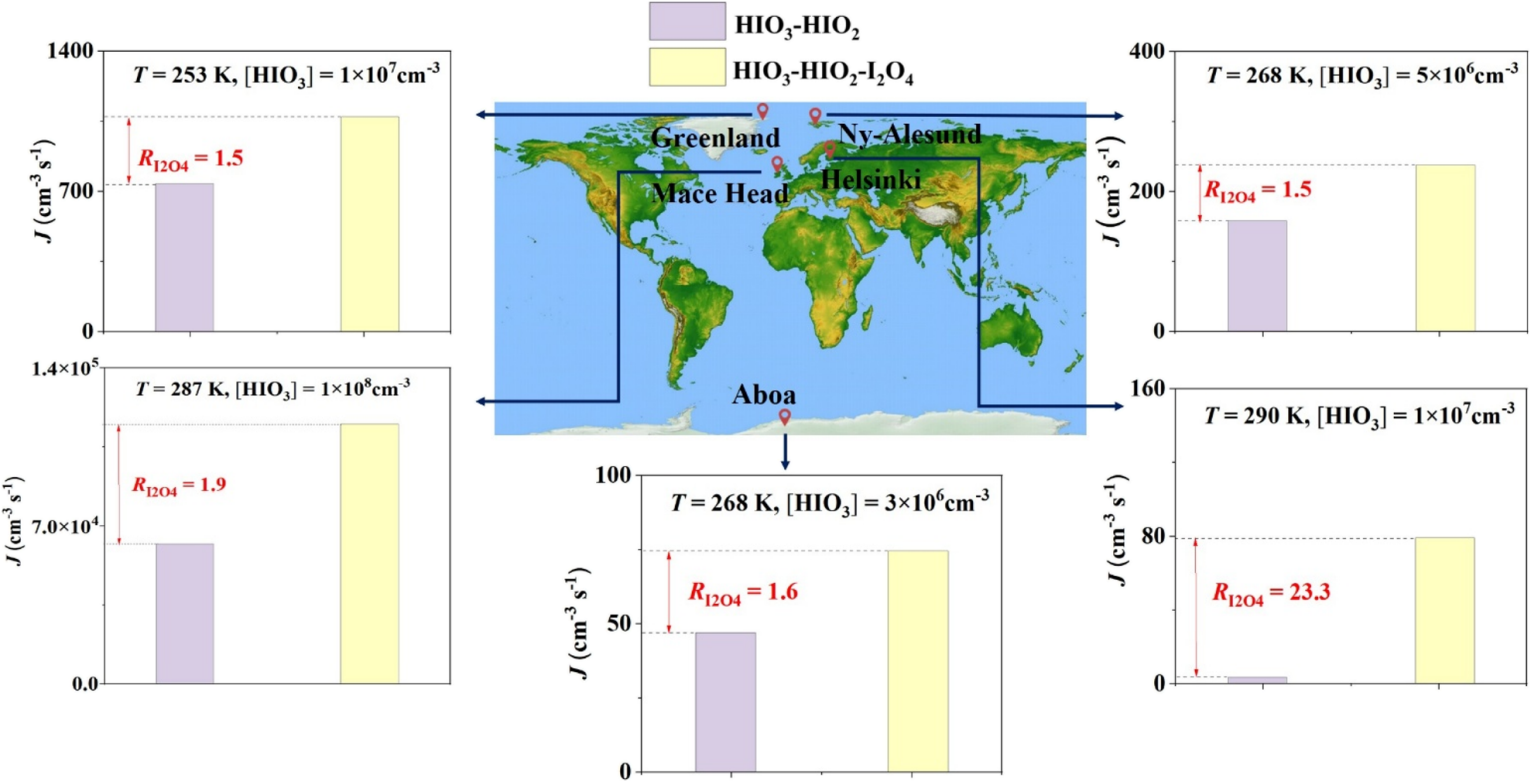
Cluster formation rate (*J*) (cm^−3^ s^−1^) of the HIO_3_–HIO_2_–I_2_O_4_ and HIO_3_–HIO_2_ systems and the enhancement coefficient (*R*_I_2_O_4__) in different areas with different temperatures (*T*) and [HIO_3_]. The *T* and [HIO_3_] in different areas were obtained from He *et al.*^38^ The *k*_coag_ in Helsinki and Mace Head was set to 2 × 10^−3^ s^−1^,^55^ and *k*_coag_ in Aboa, Greenland, and Ny-Ålesund was set to 1 × 10^−4^ s^−1^.^38^

This study reveals that I_2_O_4_, HIO_3_ and HIO_2_ can nucleate together, breaking through the previous findings that iodine oxoacids and iodine oxides independently nucleate.^14,38^ More importantly, I_2_O_4_, HIO_3_ and HIO_2_ nucleate in a synergistic way. This synergistic nucleation increases the contribution of iodine-containing species to NPF beyond what was previously thought. Fig. 6 shows the significance of HIO_3_–HIO_2_–I_2_O_4_ nucleation on a global scale. It is noted that HIO_3_–HIO_2_ system was selected as the base case, since HIO_3_–HIO_2_ is the only two-component system that was verified to have high nucleation rate at the realistic atmospheric concentration ranges of HIO_2_ and HIO_3_ (ref. 38) and has the highest nucleation rate among three two-component systems in most conditions. As shown in Fig. 6, the nucleation rate increased by at least 1.5 times in areas where HIO_3_ was detected once I_2_O_4_ was involved in nucleation. Under conditions of relatively high temperature and 10^7^ cm^−3^ HIO_3_ in Helsinki, the nucleation rate can be increased by up to 23.3 times. In addition, I_2_O_4_ enhances HIO_3_–HIO_2_ nucleation as a base. Atmospheric acids such as SA and methanesulfonic acid (MSA) have been proposed to enhance HIO_3_–HIO_2_ nucleation.^25,26,64,65^ Therefore, multicomponent nucleation involving HIO_2_, HIO_3_, I_2_O_4_, SA, MSA and other acids can further increase the contribution of iodine-containing species to NPF, which deserves future ambient field and laboratory study.

This study revealed that XBs can induce the basicity enhancement of I_2_O_4_ and HIO_2_, further driving their acid–base reactions and therefore facilitating nucleation. Since the formation of XBs is common in iodine and oxygen-containing clusters, the mechanism by which XBs induce basicity enhancement could also be extended to other nucleation systems containing iodine oxoacids and iodine oxides. We found that XBs even can induce the basicity enhancement of more acidic HIO_3_ in our preliminary study. Therefore, the mechanism by which XBs induce basicity enhancement is expected to be a common mechanism in nucleation systems containing iodine oxoacids and iodine oxides, necessitating further study. In addition, XB-induced basicity enhancement is also expected to occur during the growth of iodine oxoacids and iodine oxides-containing particles. However, more species and more complicated interactions should be involved in particle growth. The possible role of such a mechanism in particle growth is worthy of further investigation.

## Conclusions

In this study, quantum chemical methods and ACDC were employed to investigate the nucleation mechanism and kinetics of iodine oxides and iodine oxoacids. Of all iodine oxides, I_2_O_4_ was found to have the strongest nucleation potential towards HIO_3_–HIO_2_. I_2_O_4_ can synergistically nucleate with HIO_3_–HIO_2_, breaking through the previous findings that iodine oxoacids and iodine oxides independently nucleate.^14,38^ The synergistic nucleation rate of HIO_3_–HIO_2_–I_2_O_4_ is 1.5 to 6.8 times higher than that of the known most efficient iodine-associated two-component (HIO_3_–HIO_2_) nucleation at 278.15 K. The high synergistic nucleation rate enhances the role of iodine-containing species in marine atmospheric particle formation. Microscopic analysis of the three-component cluster configurations revealed that an unexpected acid–base reaction between I_2_O_4_ and HIO_2_/HIO_3_ is a key driver of this efficient synergistic nucleation, in addition to traditional HBs and XBs. The halogen bond-induced basicity enhancement was further identified as the chemical nature of I_2_O_4_ behaving as a base in the nucleation with HIO_2_ or HIO_3_. Such a basicity enhancement effect can be extended to other iodine-containing species, *e.g.*, HIO_2_ and even more acidic HIO_3_, suggesting that this is a common feature in interactions between iodine-containing species. Our findings clarify the synergistic nucleation of iodine oxoacids and iodine oxides and highlight the importance of halogen bond-induced basicity enhancement in the formation of iodine-containing particles.

## Author contributions

H. B. X. and J. S. F. designed the study. R. J. Z. and Y. Y. L. performed the quantum chemical calculation and analyzed data. R. J. Z., Y. Y. L., R. J. Y., F. F. M., H. B. X. and J. S. F. wrote the manuscript. D. M. X., J. W. C., H. B. X. and J. S. F. commented on and revised the manuscript. All coauthors participated in relevant scientific discussion of the manuscript.

## Conflicts of interest

The authors declare no competing financial interest.

## Supplementary Material

SC-OLF-D5SC02517F-s001

## Data Availability

The data supporting this article have been included as part of the ESI.†
