# Supplementary material for: Significance of halogen bonding in the synergistic nucleation of iodine oxoacids and iodine oxides
Source: Chem Sci. 2025 Jul 21;16(35):15935–46. doi: 10.1039/d5sc02517f (PMC12352695; doi:10.1039/d5sc02517f)
Supplement: SC-016-D5SC02517F-s001 [file SC-016-D5SC02517F-s001.pdf]

Supplementary Information (SI) for Chemical Science.
This journal is © The Royal Society of Chemistry 2025

Supporting Information

Significance of Halogen Bonding in the Synergistic Nucleation of Iodine Oxoacids and Iodine Oxides

Rongjie Zhang[1#], Yueyang Liu[1#], Rujing Yin[1], Fangfang Ma[1], Deming Xia[1,2], Jingwen Chen[1], Hong-Bin Xie[1*], and Joseph S. Francisco[2*]

[1]Key Laboratory of Industrial Ecology and Environmental Engineering (Ministry of Education), School of Environmental Science and Technology, Dalian University of Technology, Dalian 116024, China

[2]Department of Earth and Environmental Science, University of Pennsylvania, Philadelphia, Pennsylvania 19104-6316, United States

[#]Rongjie Zhang and Yueyang Liu contributed equally.

*Corresponding email: hbxie@dlut.edu.cn; frjoseph@sas.upenn.edu

Totally, 40 pages, 10 figures, 8 tables

Supporting Information

Contents

Selection of Boundary Clusters. 3

Selection for The Concentration of I_2O_4 3

Computational Methods for Δ*ESP*. 4

Computational Methods for Cluster Formation Rate. 4

Table S1 5

Table S2 6

Table S3 7

Table S4 8

Table S5 9

Table S6 10

Table S7 12

Table S8 14

Figure S1. 15

Figure S2. 15

Figure S3. 16

Figure S4. 17

Figure S5. 18

Figure S6. 19

Figure S7. 20

Figure S8. 21

Figure S9. 22

Figure S10. 23

References 24

Cartesian coordinates of the global minimum configurations 25

Supporting Information

Selection of Boundary Clusters.

The boundary clusters are allowed to leave the simulation box for further growth, which are required to have favorable compositions for high stability (low evaporation rate).[1] As shown in the Figure S7, $(HIO_3)_2(I_2O_4)_3$, $(HIO_3)_3(I_2O_4)_2$, $(HIO_3)_3(I_2O_4)_3$, $(HIO_2)_2(I_2O_4)_3$, $(HIO_3)_2(HIO_2)_3$, $(HIO_3)_3(HIO_2)_2$, $(HIO_3)_3(HIO_2)_3$, $(HIO_3)_3(I_2O_4)_1(HIO_2)_1$, $(HIO_3)_2(I_2O_4)_1(HIO_2)_2$, $(HIO_3)_3(I_2O_4)_1(HIO_2)_2$ and $(HIO_3)_3(I_2O_4)_2(HIO_2)_1$ clusters have evaporation rates on the order of 10^{-3} s^{-1} to 10^{-11} s^{-1} at 278.15 K, 1atm. Hence, their relevant clusters $(HIO_3)_3(I_2O_4)_4$, $(HIO_3)_4(I_2O_4)_3$, $(HIO_3)_4(I_2O_4)_4$, $(HIO_2)_3(I_2O_4)_4$, $(HIO_3)_3(HIO_2)_4$, $(HIO_3)_4(HIO_2)_3$, $(HIO_3)_4(HIO_2)_4$, $(HIO_3)_4(HIO_2)_1(I_2O_4)_2$, $(HIO_3)_4(HIO_2)_2(I_2O_4)_1$, $(HIO_3)_3(HIO_2)_3(I_2O_4)_1$, $(HIO_3)_3(HIO_2)_2(I_2O_4)_2$, $(HIO_3)_4(HIO_2)_1(I_2O_4)_3$, $(HIO_3)_4(HIO_2)_2(I_2O_4)_2$ and $(HIO_3)_4(HIO_2)_3(I_2O_4)_1$ are selected as boundary clusters in the ACDC simulations of HIO_3-HIO_2-I_2O_4 system at 278.15 K, 1atm.

Selection for the Concentration of I_2O_4.

I_2O_4 has been measured at steady-state concentrations of ~1% of HIO_3 under marine boundary layer conditions in the CERN CLOUD chamber.[2] This steady-state concentration of I_2O_4 arises from the balance between its formation and degradation reaction. Recent flow tube experiments also found that I_2O_4 owns enough steady-state concentration to nucleate with other iodine-containing species even though relatively faster hydrolysis of I_2O_4 occurs,[3] indicating that hydrolysis reaction can't "consume" all I_2O_4. Therefore, when low steady-state concentration of I_2O_4 was employed in the ACDC simulations (1% of $[HIO_3]$), it could be unnecessary to consider the transformation reaction as a competing process.

Supporting Information

Computational Methods for ΔESP.

The change in electrostatic potential (ΔESP) value (kcal mol^{-1}) of a specific atom is calculated by the following formula:

ΔESP = ESP (a specific atom in dimer) – ESP (the same atom in monomer) (1)

where the dimer is monomer-precursor dimer cluster formed by halogen bond or hydrogen bond. Multiwfn (Version 3.7) is employed to obtain the electrostatic potential value (ESP) for a specific atom.[4] The evaluation of ESP by Multiwfn is based on an efficient algorithm, which regroups the *ESP* expression in terms of primitive Gaussian type orbitals (GTOs), and then each type of contribution in the expression is calculated using a computerized optimized code.[5]

Computational Methods for Cluster Formation Rate.

The cluster formation rate (J) (cm^{-3} s^{-1}) is defined as the flux of clusters outside the system including all the boundary clusters and any greater clusters. J is calculated as the following formula in ACDC:

$$J = \sum_{i} \frac{1}{2} \sum_{j<i} \beta_{j,(i-j)} \, c_j c_{(i-j)} \qquad (2)$$

where i is the boundary cluster and any greater cluster of the system, j is the cluster in the system which can collide with another cluster or monomer ($i-j$) to form cluster i, $\beta_{j,(i-j)}$ is the collision coefficient between clusters j and i-j, c_j is the number concentration of cluster j.

Supporting Information

Table S1. Comparison of Δ*G* (kcal mol^{-1}) for $(HIO_3)_{1\text{-}3}(HIO_2)_{1\text{-}3}$ clusters calculated at method 1 (DLPNO-CCSD(T)/aug-cc-pVTZ(-PP)//M06-2X/6-31++G(d,p) + aug-cc-pVTZ-PP) and method 2 (DLPNO-CCSD(T)/aug-cc-pVTZ(-PP)//M06-2X/aug-cc-pVTZ(-PP)) at 298.15 K with quasi-harmonic correction.[6]

Clusters	Δ*G* (method 1)	Δ*G* (method 2)	biases
$(HIO_3)_1(HIO_2)_1$	-16.65	-16.52	0.13
$(HIO_3)_2$	-9.73	-9.46	0.27
$(HIO_2)_2$	-17.69	-17.42	0.27
$(HIO_3)_1(HIO_2)_2$	-34.39	-34.45	-0.06
$(HIO_3)_2(HIO_2)_1$	-30.02	-29.93	0.09
$(HIO_3)_3$	-18.20	-18.03	0.17
$(HIO_2)_3$	-33.69	-33.49	0.20
$(HIO_3)_2(HIO_2)_2$	-46.72	-46.90	-0.18
$(HIO_3)_3(HIO_2)_1$	-40.92	-41.29	-0.37
$(HIO_3)_1(HIO_2)_3$	-47.66	-47.68	-0.02
$(HIO_3)_3(HIO_2)_2$	-62.43	-62.65	-0.22
$(HIO_3)_2(HIO_2)_3$	-64.77	-64.86	-0.09
$(HIO_3)_3(HIO_2)_3$	-86.86	-87.05	-0.19

Supporting Information

Table S2. The structural root-mean-square deviation (RMSD) (Å) and final Δ*G* values (kcal mol^{-1}) of cluster global minima with and without dispersion correction.

Clusters	RMSD	Δ*G* with dispersion correction	Δ*G* without dispersion correction	Δ*G* difference
HIO_3	1.6×10^{-7}	–	–	–
HIO_2	2×10^{-7}	–	–	–
I_2O_4	0.0011	–	–	–
$(HIO_2)_1(I_2O_4)_1$	0.002	-21.6104	-21.6086	-0.0018
$(HIO_3)_2(I_2O_4)_2$	0.0014	-57.0182	-57.03	0.0118
$(HIO_3)_1(HIO_2)_1(I_2O_4)_1$	0.0032	-38.9885	-38.9845	-0.004

Supporting Information

Table S3. Average energy gap (kcal mol^{-1}) between antibonding orbital δ*(O−I) and lone-pair orbital LP(O), which are two critical molecular orbitals for forming two halogen bonds (XBs) between I_xO_y and HIO_2.

I_xO_y-HIO_2 dimer	XB type	Energy gap	Average energy gap
I_2O_4-HIO_2 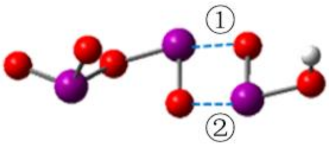	XB ①<br>XB ②	194.47<br>225.59	210.03
I_2O_2-HIO_2 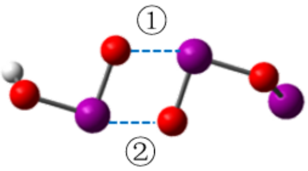	XB ①<br>XB ②	216.43<br>218.81	217.62
I_2O_3-HIO_2 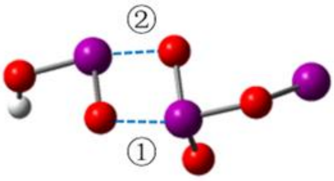	XB ①<br>XB ②	203.19<br>239.27	221.26
I_2O_5-HIO_2 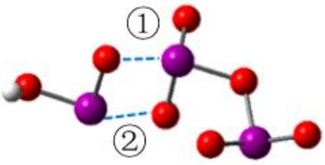	XB ①<br>XB ②	181.85<br>254.64	218.25

Supporting Information

Table S4. The identity of selected precursors for forming I_2O_4-precursor dimers with halogen bond (XB) type 1 and type 2, the corresponding XB energy (kcal mol^{-1}) and the change in electrostatic potential (ΔESP) (kcal mol^{-1}) for the O-atom 1 and 2 of I_2O_4 when it forms XBs with precursors.

Abbreviations of precursors	Common names of precursors	XB type 1		XB type 2	
		energy	ΔESP	energy	ΔESP
DMA	dimethylamine	23.65	-16.31	25.78	-22.37
ForM	formaldehyde	12.34	-8.25	12.61	-12.83
MeSH	methanethiol	12.25	-9.45	13.30	-16.33
DMS	dimethyl sulfide	16.45	-12.05	19.29	-19.27
TMA	trimethylamine	26.83	-16.99	28.98	-22.74
DEA	diethylamine	24.08	-16.37	28.08	-22.58
TEA	triethylamine	27.55	-17.57	——	——
AA	acetaldehyde	14.35	-10.9	14.75	-15.75
Gly	glyoxal	10.82	-5.84	11.23	-5.63
MA	methylamine	19.76	-15.1	21.80	-20.71
EA	ethanamine	20.21	-15.37	22.43	-21.11
AEA	aminomethanol	17.09	-12.65	19.29	-18.39
MEA	ethanolamine	20.33	-15.32	22.59	-21.41
EDA	ethylenediamine	23.18	-16.86	25.50	-23.07
AC	acetone	15.74	-12.23	15.40	-16.64
Mgly	methylglyoxal	12.16	-6.78	11.58	-11.16
MEK	2-butanone	16.23	-12.51	16.03	-17.04
BT	benzothiazole	16.48	-14.46	18.90	-20.83
MHP	methyl hydroperoxide	13.01	-9.67	11.70	-13.06
MO	methanol	——	——	12.85	-12.98
HPMTF	hydroperoxymethyl thioformate	13.10	-9.24	13.47	-13.72

Table S5. The change in electrostatic potential (ΔESP) values (kcal mol^{-1}) for the N atom of NH_3 when it forms hydrogen bonds (HBs) with precursors and the corresponding HB energy (kcal mol^{-1}).

Dimers	ΔESP for the N atom of NH_3	HB energy
TMA-NH_3	-2.11	4.56
TEA-NH_3	-5.83	4.72
AC-NH_3	-0.14	4.32
Gly-NH_3	-1.10	1.63

Supporting Information

Table S6. Formation free energies (Δ*G*, kcal mol^{-1}) of HIO_3-HIO_2-I_2O_4 system clusters calculated at DLPNO-CCSD(T)/aug-cc-pVTZ(-PP)//M06-2X/aug-cc-pVTZ(-PP) level of theory at different temperatures (K) and 1atm.

Clusters	298.15 K	278.15 K	253 K	223.15 K
$(HIO_2)_2$	-17.93	-18.77	-19.84	-21.11
$(HIO_2)_3$	-34.50	-36.21	-38.38	-40.96
$(I_2O_4)_2$	-24.24	-25.14	-26.29	-27.65
$(I_2O_4)_3$	-41.69	-43.75	-46.36	-49.47
$(HIO_3)_2$	-9.18	-10.17	-11.41	-12.90
$(HIO_3)_3$	-18.36	-20.25	-22.64	-25.49
$(HIO_3)_1(HIO_2)_1$	-16.79	-17.69	-18.83	-20.18
$(HIO_3)_2(HIO_2)_1$	-29.90	-31.82	-34.25	-37.15
$(HIO_3)_3(HIO_2)_1$	-43.10	-45.82	-49.25	-53.33
$(HIO_3)_1(HIO_2)_2$	-35.75	-37.52	-39.75	-42.41
$(HIO_3)_2(HIO_2)_2$	-49.15	-51.78	-55.11	-59.08
$(HIO_3)_3(HIO_2)_2$	-64.98	-68.66	-73.30	-78.83
$(HIO_3)_1(HIO_2)_3$	-49.60	-52.28	-55.64	-59.65
$(HIO_3)_2(HIO_2)_3$	-67.84	-71.37	-75.83	-81.15
$(HIO_3)_3(HIO_2)_3$	-89.42	-94.07	-99.95	-106.95
$(HIO_3)_1(I_2O_4)_1$	-18.01	-21.42	-20.02	-21.36
$(HIO_3)_2(I_2O_4)_1$	-33.29	-35.25	-37.72	-40.67
$(HIO_3)_3(I_2O_4)_1$	-50.24	-53.24	-57.00	-61.44
$(HIO_3)_1(I_2O_4)_2$	-41.07	-43.18	-45.84	-49.01
$(HIO_3)_2(I_2O_4)_2$	-57.03	-60.23	-64.28	-69.10
$(HIO_3)_3(I_2O_4)_2$	-72.78	-77.18	-82.44	-88.70
$(HIO_3)_1(I_2O_4)_3$	-64.88	-71.47	-71.28	-76.46
$(HIO_3)_2(I_2O_4)_3$	-88.19	-92.42	-97.75	-104.17
$(HIO_3)_3(I_2O_4)_3$	-106.54	-109.60	-116.28	-126.14
$(HIO_2)_1(I_2O_4)_1$	-21.61	-22.51	-23.65	-25.01
$(HIO_2)_1(I_2O_4)_2$	-46.84	-48.92	-51.56	-54.69
$(HIO_2)_1(I_2O_4)_3$	-65.26	-68.29	-72.11	-76.68
$(HIO_2)_2(I_2O_4)_1$	-38.17	-40.06	-42.44	-45.27
$(HIO_2)_2(I_2O_4)_2$	-73.61	-76.61	-80.39	-84.89

$(HIO_2)_2(I_2O_4)_3$	-99.31	-103.46	-108.70	-114.94
$(HIO_2)_3(I_2O_4)_1$	-54.38	-57.20	-60.75	-64.99
$(HIO_2)_3(I_2O_4)_2$	-81.72	-85.53	-90.33	-96.06
$(HIO_2)_3(I_2O_4)_3$	-114.48	-119.44	-125.69	-133.15
$(HIO_3)_1(HIO_2)_1(I_2O_4)_1$	-38.98	-40.89	-43.29	-46.15
$(HIO_3)_2(HIO_2)_1(I_2O_4)_1$	-50.49	-53.35	-56.96	-61.26
$(HIO_3)_3(HIO_2)_1(I_2O_4)_1$	-69.15	-72.99	-77.84	-83.63
$(HIO_3)_1(HIO_2)_1(I_2O_4)_2$	-60.35	-63.32	-67.07	-71.55
$(HIO_3)_2(HIO_2)_1(I_2O_4)_2$	-74.58	-78.59	-83.64	-89.67
$(HIO_3)_3(HIO_2)_1(I_2O_4)_2$	-94.13	-99.09	-105.36	-112.82
$(HIO_3)_1(HIO_2)_2(I_2O_4)_1$	-52.10	-54.89	-58.41	-62.60
$(HIO_3)_2(HIO_2)_2(I_2O_4)_1$	-71.61	-75.42	-80.24	-85.97
$(HIO_3)_3(HIO_2)_2(I_2O_4)_1$	-92.52	-97.27	-103.27	-110.42

Supporting Information

Table S7. Formation free energies (Δ*G*, kcal mol^{-1}) of HIO_3-HIO_2-I_2O_4 system clusters calculated at DLPNO-CCSD(T)/aug-cc-pVTZ(-PP)//M06-2X/aug-cc-pVTZ(-PP) level of theory at different atmospheric pressures (atm) and 278.15 K.

Clusters	1 atm	0.5 atm	0.1 atm
$(HIO_2)_2$	-18.77	-18.39	-17.50
$(HIO_2)_3$	-36.21	-35.44	-33.67
$(I_2O_4)_2$	-25.14	-24.76	-23.87
$(I_2O_4)_3$	-43.75	-42.99	-41.21
$(HIO_3)_2$	-10.17	-9.78	-8.90
$(HIO_3)_3$	-20.25	-19.49	-17.71
$(HIO_3)_1(HIO_2)_1$	-17.69	-17.31	-16.42
$(HIO_3)_2(HIO_2)_1$	-31.82	-31.06	-29.28
$(HIO_3)_3(HIO_2)_1$	-45.82	-44.67	-42.01
$(HIO_3)_1(HIO_2)_2$	-37.52	-36.75	-34.98
$(HIO_3)_2(HIO_2)_2$	-51.78	-50.64	-47.97
$(HIO_3)_3(HIO_2)_2$	-68.66	-67.13	-63.58
$(HIO_3)_1(HIO_2)_3$	-52.28	-51.13	-48.46
$(HIO_3)_2(HIO_2)_3$	-71.37	-69.84	-66.29
$(HIO_3)_3(HIO_2)_3$	-94.07	-92.16	-87.72
$(HIO_3)_1(I_2O_4)_1$	-21.42	-18.51	-17.63
$(HIO_3)_2(I_2O_4)_1$	-35.25	-34.48	-32.71
$(HIO_3)_3(I_2O_4)_1$	-53.24	-52.07	-49.41
$(HIO_3)_1(I_2O_4)_2$	-43.18	-42.42	-40.64
$(HIO_3)_2(I_2O_4)_2$	-60.23	-59.09	-56.42
$(HIO_3)_3(I_2O_4)_2$	-77.18	-75.64	-72.09
$(HIO_3)_1(I_2O_4)_3$	-71.47	-66.81	-64.14
$(HIO_3)_2(I_2O_4)_3$	-92.42	-91.02	-87.47
$(HIO_3)_3(I_2O_4)_3$	-109.60	-109.83	-105.39
$(HIO_2)_1(I_2O_4)_1$	-22.51	-22.13	-21.24
$(HIO_2)_1(I_2O_4)_2$	-48.92	-48.16	-46.38
$(HIO_2)_1(I_2O_4)_3$	-68.29	-67.14	-64.48
$(HIO_2)_2(I_2O_4)_1$	-40.06	-39.29	-37.52
$(HIO_2)_2(I_2O_4)_2$	-76.61	-75.46	-72.80
$(HIO_2)_2(I_2O_4)_3$	-103.46	-101.93	-98.38
$(HIO_2)_3(I_2O_4)_1$	-57.20	-56.05	-53.39
$(HIO_2)_3(I_2O_4)_2$	-85.53	-83.99	-80.45
$(HIO_2)_3(I_2O_4)_3$	-119.44	-117.52	-113.09
$(HIO_3)_1(HIO_2)_1(I_2O_4)_1$	-40.89	-40.12	-38.35
$(HIO_3)_2(HIO_2)_1(I_2O_4)_1$	-53.35	-52.20	-49.54
$(HIO_3)_3(HIO_2)_1(I_2O_4)_1$	-72.99	-71.46	-67.91
$(HIO_3)_1(HIO_2)_1(I_2O_4)_2$	-63.32	-62.17	-59.51

$(HIO_3)_2(HIO_2)_1(I_2O_4)_2$	-78.59	-77.05	-73.51
$(HIO_3)_3(HIO_2)_1(I_2O_4)_2$	-99.09	-97.18	-92.74
$(HIO_3)_1(HIO_2)_2(I_2O_4)_1$	-54.89	-53.74	-51.08
$(HIO_3)_2(HIO_2)_2(I_2O_4)_1$	-75.42	-73.89	-70.34
$(HIO_3)_3(HIO_2)_2(I_2O_4)_1$	-97.27	-95.36	-90.92

Table S8. Formation free energies (Δ*G*, kcal mol^{-1}) at 298.15 K of organic acids (benzoic acid, cis-pinonic acid, formic acid)-NH_3,[7] I_2O_4-HIO_2 and HIO_2-HIO_2 dimers.[6]

Bond type	Dimers	Δ*G*
Hydrogen bond	benzoic acid-NH_3	-0.54
	cis-pinonic acid-NH_3	0.20
	formic acid-NH_3	-0.26
Halogen bond	I_2O_4-HIO_2	-21.6
	HIO_2-HIO_2	-17.93

Supporting Information

Cluster	M062X/6-31++G(d,p) + aug-cc-pvtz-pp			M062X/def2tzvp		
	Configuration Name	*G* (Hartee)	*G* difference with the lowest-energy configuration (kcal mol^{-1})	Configuration Name	*G* (Hartee)	*G* difference with the lowest-energy configuration (kcal mol^{-1})
$(HIO_3)_1(HIO_2)_1(I_2O_4)_1$	371.log	-1859.9224	0.00	Con-8_0_76.log	-1868.2533	0.00
	Con-8_0_76.log	-1859.9220	0.26	371.log	-1868.2529	0.24
	Con-2_3_184.log	-1859.9200	1.51	Con-2_3_184.log	-1868.2498	2.20
	Con-8_2_126.log	-1859.9170	3.40	Con-8_2_126.log	-1868.2478	3.42
	211.log	-1859.9160	4.03	211.log	-1868.2458	4.67
	Con-8_2_3.log	-1859.9149	4.69	Con-8_2_236.log	-1868.2448	5.31
	Con-8_2_236.log	-1859.9149	4.74	Con-8_2_3.log	-1868.2447	5.37
	1440.log	-1859.9142	5.16	1440.log	-1868.2441	5.77
	536.log	-1859.9141	5.24	536.log	-1868.2438	5.91
	Con-3_0_121.log	-1859.9135	5.60	Con-3_0_121.log	-1868.2436	6.04
$(HIO_2)_1(I_2O_4)_1$	Con-4_3_79.log	-1338.2628	0.00	Con-4_3_79.log	-1344.4827	0.00
	Con-4_0_46.log	-1338.2557	4.47	Con-4_0_46.log	-1344.4771	3.53
	2417.log	-1338.2548	5.03	2417.log	-1344.4753	4.67
	Con-4_0_227.log	-1338.2538	5.66	Con-4_0_227.log	-1344.4737	5.66
	Con-4_2_203.log	-1338.2537	5.76	Con-4_2_203.log	-1344.4734	5.84
$(HIO_3)_2(I_2O_4)_2$	Con-1_2_239.log	-2826.8304	0.00	Con-1_2_239.log	-2839.3461	0.00
	Con-4_0_9.log	-2826.8236	4.25	Con-4_0_9.log	-2839.3400	3.80
	Con-4_0_67.log	-2826.8228	4.74	1508.log	-2839.3382	4.93
	1508.log	-2826.8210	5.87	2835.log	-2839.3374	5.45
	2835.log	-2826.8204	6.30	Con-4_1_111.log	-2839.3371	5.60
	1195.log	-2826.8203	6.35	1195.log	-2839.3371	5.60
	——	——	——	Con-4_0_67.log	-2839.3366	5.94

Figure S1. Energy sorting of the configurations (within ~6 kcal mol^{-1} of the lowest-energy configuration) after optimization at method 1 (M062X/6-31++G (d,p) + aug-cc-pVTZ-PP) and method 2 (M062X/def2TZVP). The configuration names in red indicate the low-energy configurations (within 2 kcal mol^{-1} of the lowest-energy one).

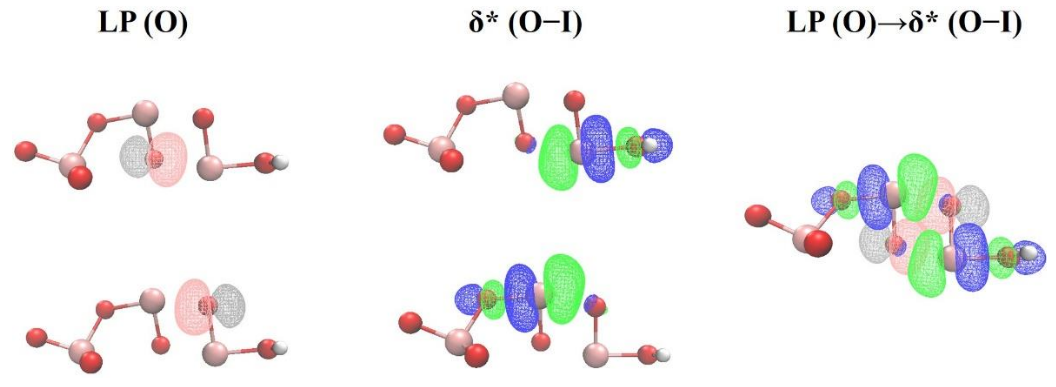

Figure S2. The natural bond orbitals (NBOs) for the formed halogen bonds in the $(I_2O_4)_1(HIO_2)_1$ dimer cluster. LP(O) indicates the lone-pair orbitals, and δ*(O−I) indicates the antibonding orbitals. The orbitals are visualized via Multiwfn 3.7[4] and VMD 1.9.3.[8]

Supporting Information

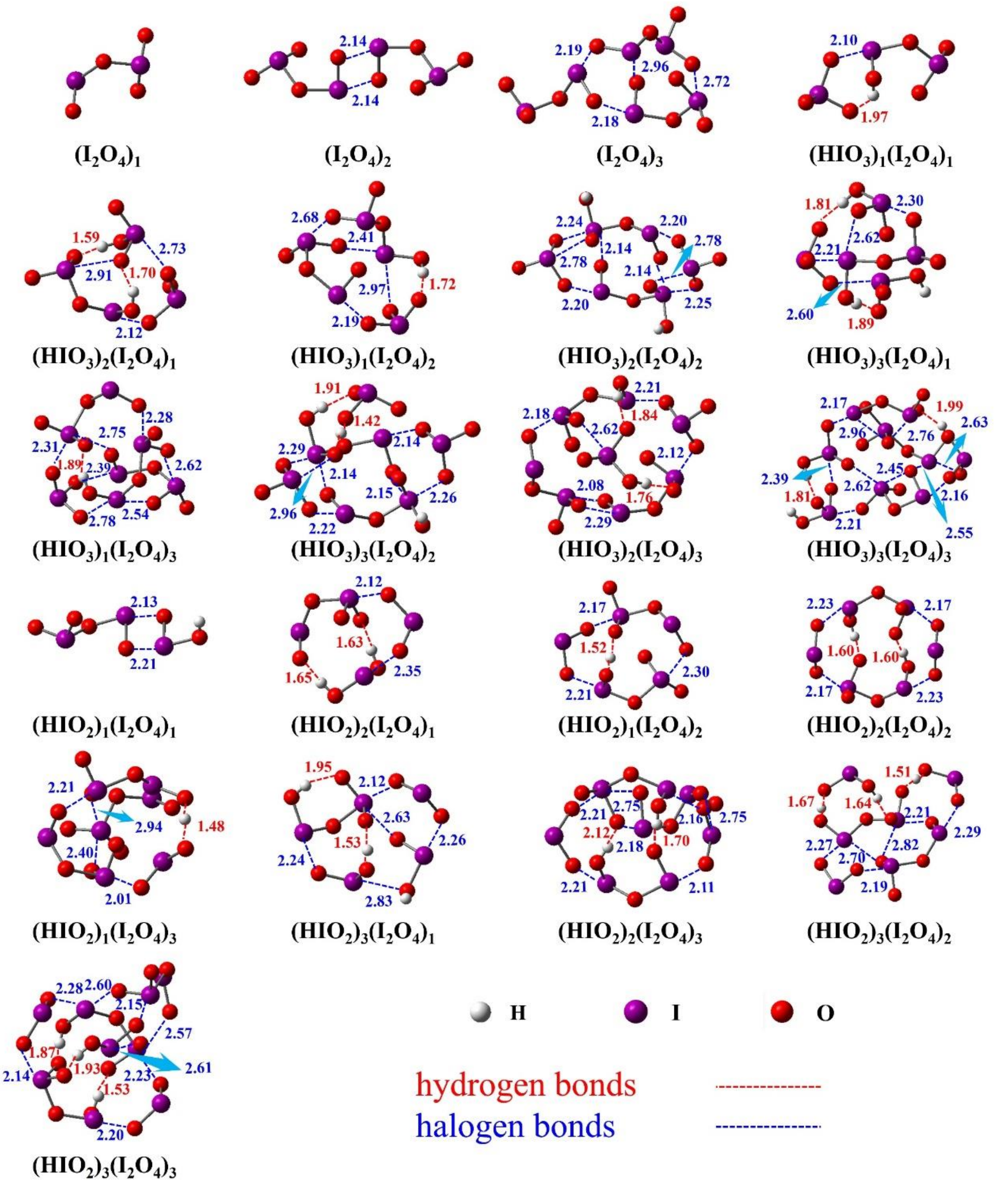

Figure S3. Global minimum configurations of the $(HIO_3)_{0\text{-}3}(I_2O_4)_{1\text{-}3}$ and $(HIO_2)_{1\text{-}3}(I_2O_4)_{1\text{-}3}$ clusters calculated at the DLPNO-CCSD(T)/aug-cc-pVTZ(-PP)//M06-2X/aug-cc-pVTZ(-PP) level of theory. The red and blue numbers indicate the bond lengths of HBs and XBs, respectively. The bond lengths are given in Å.

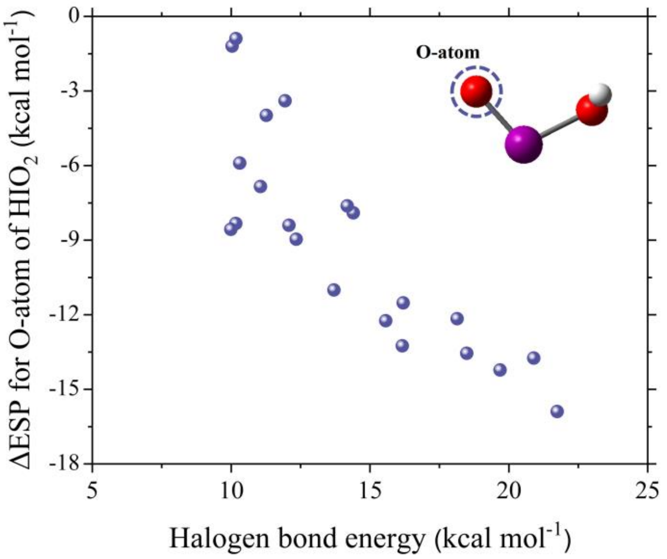

Figure S4. Change in electrostatic potential (ΔESP) values (kcal mol^{-1}) for the terminal O atom as a basic site of HIO_2 when it forms halogen bond with precursors as a function of the halogen bond energy (kcal mol^{-1}).

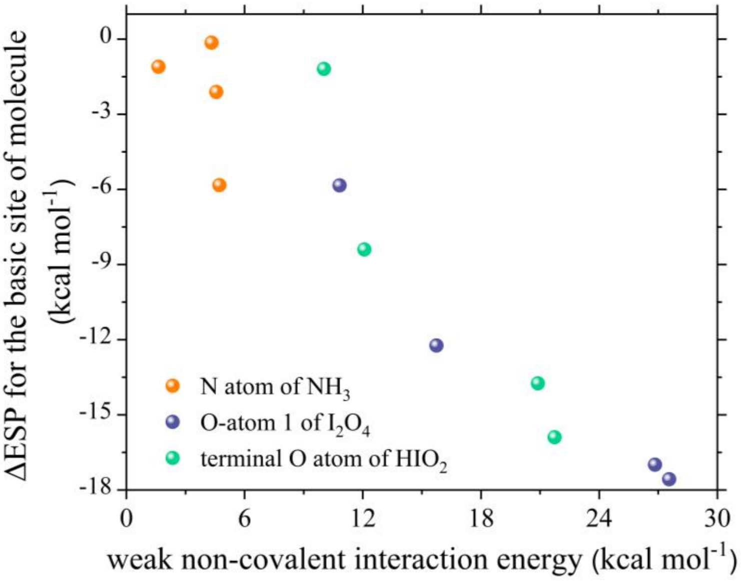

Figure S5. Change in electrostatic potential (ΔESP) values (kcal mol^{-1}) for the basic site of molecules (N atom of NH_3 (orange spheres), O-atom 1 of I_2O_4 (purple spheres) and terminal O atom of HIO_2 (green spheres)) when they form hydrogen bonds (NH_3) or halogen bonds (I_2O_4 and HIO_2) with precursors as a function of the weak non-covalent interaction (hydrogen bond or halogen bond) energy (kcal mol^{-1}). The tested precursors include TMA, TEA, AC and Gly.

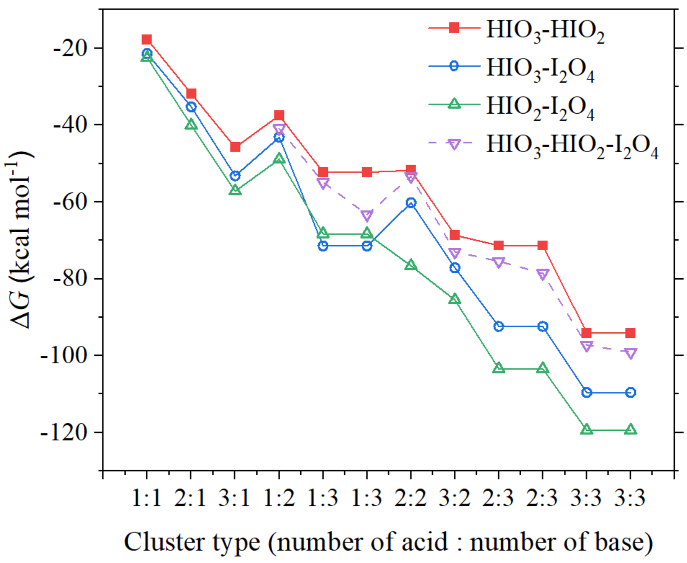

Figure S6. Formation free energy (Δ*G*) of the $(HIO_3)_{1\text{-}3}(I_2O_4)_{1\text{-}3}$ (hollow blue circles), $(HIO_2)_{1\text{-}3}(I_2O_4)_{1\text{-}3}$ (hollow green triangles), $(HIO_3)_{1\text{-}3}(HIO_2)_{1\text{-}3}$[6] (filled red squares) and $(HIO_3)_x(HIO_2)_y(I_2O_4)_z$ ($x = 1\text{-}3$, $y + z = 2\text{-}3$) clusters (hollow purple inverted triangles) calculated at the DLPNO-CCSD(T)/aug-cc-pVTZ(-PP)//M06-2X/aug-cc-pVTZ(-PP) level of theory. The calculations are performed at 278.15 K and 1 atm. Acid refers to HIO_3 for HIO_3-HIO_2, HIO_3-I_2O_4, HIO_3-HIO_2-I_2O_4 clusters, and HIO_2 for HIO_2-I_2O_4 clusters, respectively. Base refers to HIO_2 for HIO_3-HIO_2 clusters, I_2O_4 for HIO_3-I_2O_4 and HIO_2-I_2O_4 clusters, HIO_2 and I_2O_4 for HIO_3-HIO_2-I_2O_4 clusters, respectively. Repeated "1:3, 2:3, 3:3" of X-axis represent two different base combination types for three-component HIO_3-HIO_2-I_2O_4 clusters.

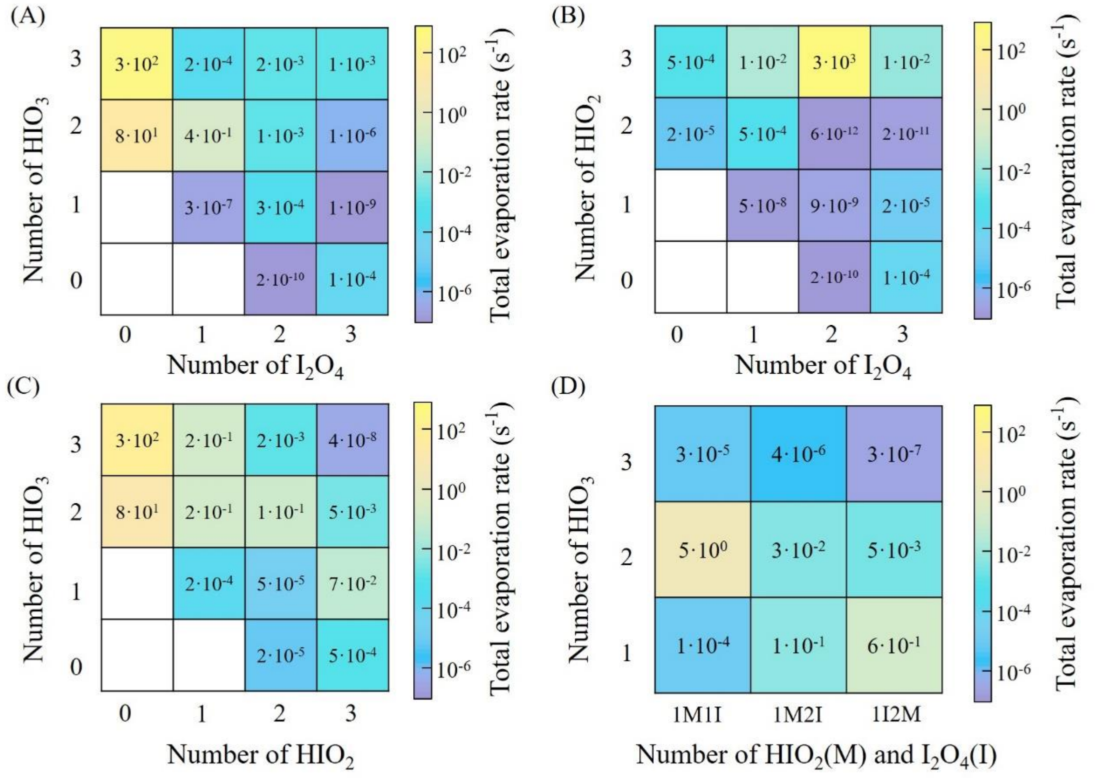

Figure S7. Evaporation rates of the $(HIO_3)_{1\text{-}3}(I_2O_4)_{1\text{-}3}$ (A), $(HIO_2)_{1\text{-}3}(I_2O_4)_{1\text{-}3}$ (B), $(HIO_3)_{1\text{-}3}(HIO_2)_{1\text{-}3}$[6] (C) and $(HIO_3)_x(HIO_2)_y(I_2O_4)_z$ (x = 1-3, $y + z$ = 2-3) (D) clusters at 278.15 K and 1 atm.

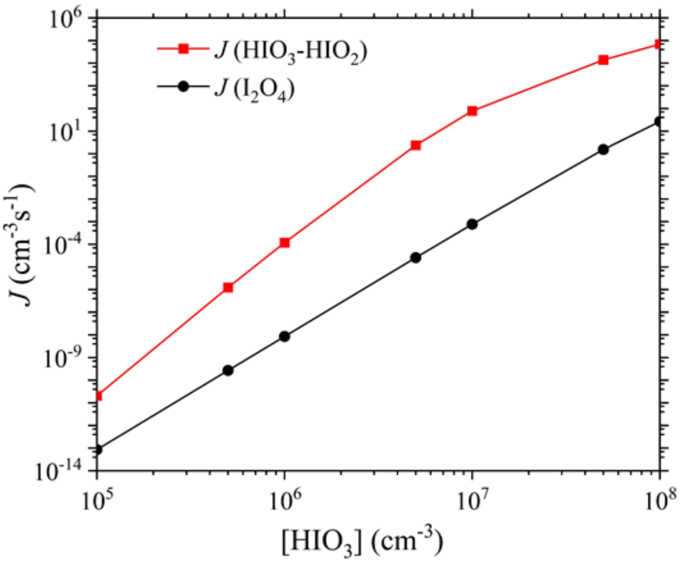

Figure S8. Cluster formation rate (J) (cm^{-3} s^{-1}) of HIO_3-HIO_2 and pure-I_2O_4 systems as a function of [HIO_3] ([HIO_2] = 1/30 [HIO_3], [I_2O_4] = 1/100 [HIO_3]) at 278.15 K, 1 atm and $k_{coag} = 0.002$ s^{-1}.

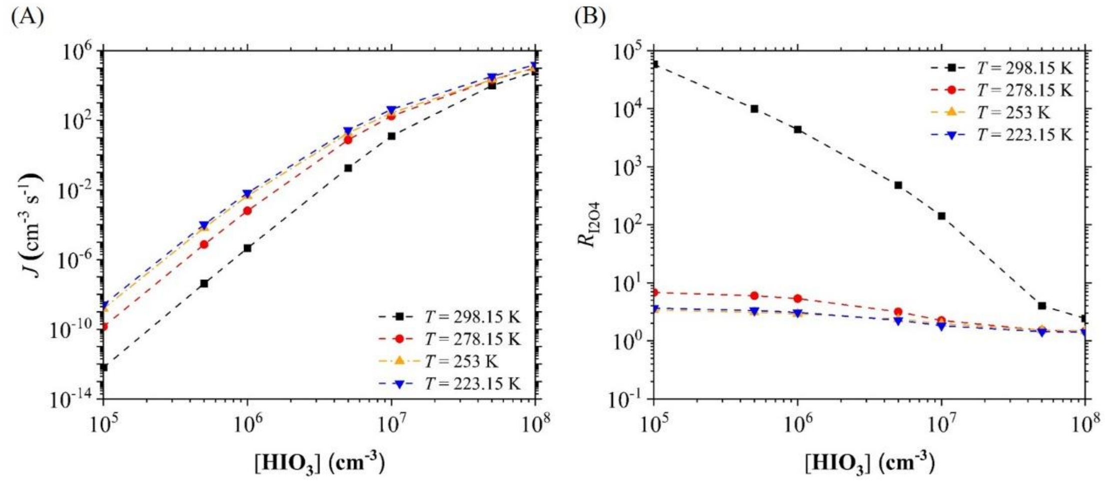

Figure S9. Cluster formation rate (J) (cm^{-3} s^{-1}) of HIO_3-HIO_2-I_2O_4 system (A) and enhancement coefficient (R_{I2O4}) (B) as a function of temperatures (T = 298.15, 278.15, 253 and 223.15 K) at 1 atm, k_{coag} = 0.002 s^{-1} and [HIO_3] = 10^5 - 10^8 cm^{-3} ([HIO_2] = 1/30 [HIO_3], [I_2O_4] = 1/100 [HIO_3]).

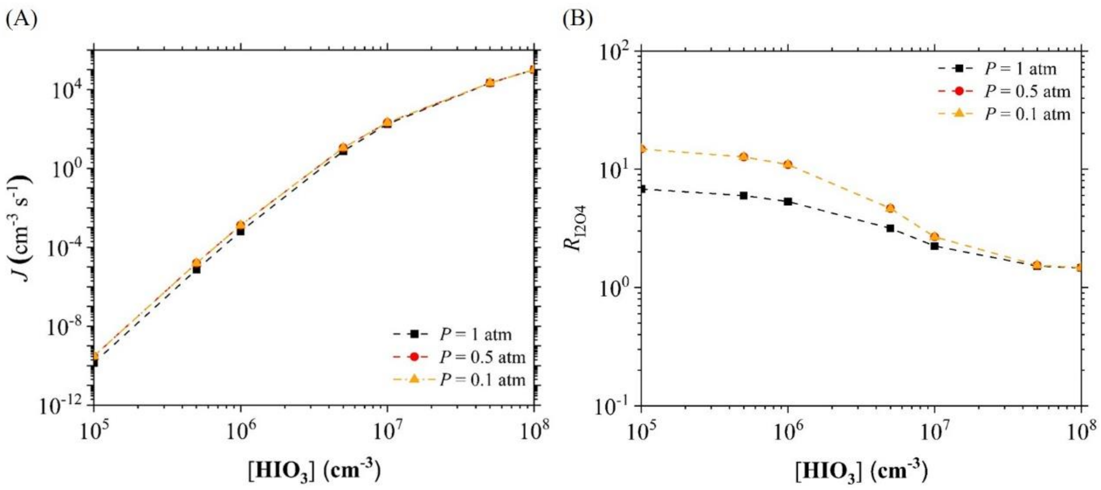

Figure S10. Cluster formation rate (J) (cm^{-3} s^{-1}) of HIO_3-HIO_2-I_2O_4 system (A) and enhancement coefficient (R_{I2O4}) (B) as a function of pressures (P = 1, 0.5 and 0.1 atm) at 278.15 K, k_{coag} = 0.002 s^{-1} and [HIO_3] = 10^5 - 10^8 cm^{-3} ([HIO_2] = 1/30 [HIO_3], [I_2O_4] = 1/100 [HIO_3]).

Supporting Information

References

1. M. J. McGrath, T. Olenius, I. K. Ortega, V. Loukonen, P. Paasonen, T. Kurtén, M. Kulmala and H. Vehkamäki, Atmospheric Cluster Dynamics Code: A Flexible Method for Solution of the Birth-Death Equations, *Atmos. Chem. Phys.*, 2012, **12**, 2345-2355.
2. X.-C. He, Y. J. Tham, L. Dada, M. Wang, H. Finkenzeller, D. Stolzenburg, S. Iyer, M. Simon, A. Kürten, J. Shen, B. Rörup, M. Rissanen, S. Schobesberger, R. Baalbaki, D. S. Wang, T. K. Koenig, T. Jokinen, N. Sarnela, L. J. Beck, J. Almeida, S. Amanatidis, A. Amorim, F. Ataei, A. Baccarini, B. Bertozzi, F. Bianchi, S. Brilke, L. Caudillo, D. Chen, R. Chiu, B. Chu, A. Dias, A. Ding, J. Dommen, J. Duplissy, I. El Haddad, L. Gonzalez Carracedo, M. Granzin, A. Hansel, M. Heinritzi, V. Hofbauer, H. Junninen, J. Kangasluoma, D. Kemppainen, C. Kim, W. Kong, J. E. Krechmer, A. Kvashin, T. Laitinen, H. Lamkaddam, C. P. Lee, K. Lehtipalo, M. Leiminger, Z. Li, V. Makhmutov, H. E. Manninen, G. Marie, R. Marten, S. Mathot, R. L. Mauldin, B. Mentler, O. Möhler, T. Müller, W. Nie, A. Onnela, T. Petäjä, J. Pfeifer, M. Philippov, A. Ranjithkumar, A. Saiz-Lopez, I. Salma, W. Scholz, S. Schuchmann, B. Schulze, G. Steiner, Y. Stozhkov, C. Tauber, A. Tomé, R. C. Thakur, O. Väisänen, M. Vazquez-Pufleau, A. C. Wagner, Y. Wang, S. K. Weber, P. M. Winkler, Y. Wu, M. Xiao, C. Yan, Q. Ye, A. Ylisirniö, M. Zauner-Wieczorek, Q. Zha, P. Zhou, R. C. Flagan, J. Curtius, U. Baltensperger, M. Kulmala, V.-M. Kerminen, T. Kurtén, N. M. Donahue, R. Volkamer, J. Kirkby, D. R. Worsnop and M. Sipilä, Role of iodine oxoacids in atmospheric aerosol nucleation, *Science*, 2021, **371**, 589-595.
3. J. C. Gómez Martín, T. R. Lewis, M. A. Blitz, J. M. C. Plane, M. Kumar, J. S. Francisco and A. Saiz-Lopez, A gas-to-particle conversion mechanism helps to explain atmospheric particle formation through clustering of iodine oxides, *Nat. Commun.*, 2020, **11**, 4521.
4. T. Lu and F. Chen, Multiwfn: A multifunctional wavefunction analyzer, *J. Comput. Chem.*, 2012, **33**, 580-592.
5. J. Zhang and T. Lu, Efficient evaluation of electrostatic potential with computerized optimized code, *Phys. Chem. Chem. Phys.*, 2021, **23**, 20323-20328.
6. R. Zhang, H.-B. Xie, F. Ma, J. Chen, S. Iyer, M. Simon, M. Heinritzi, J. Shen, Y. J. Tham, T. Kurtén, D. R. Worsnop, J. Kirkby, J. Curtius, M. Sipilä, M. Kulmala and X.-C. He, Critical Role of Iodous Acid in Neutral Iodine Oxoacid Nucleation, *Environ. Sci. Technol.*, 2022, **56**, 14166-14177.
7. J. Zhao, A. Khalizov, R. Zhang and R. McGraw, Hydrogen-Bonding Interaction in Molecular Complexes and Clusters of Aerosol Nucleation Precursors, *J. Phys. Chem. A*, 2009, **113**, 680-689.
8. W. Humphrey, A. Dalke and K. Schulten, VMD: Visual molecular dynamics, *J. Mol. Graph.*, 1996, **14**, 33-38.

Supporting Information

Cartesian coordinates of the global minimum configurations

$(I_2O_4)_1$

O	-0.06303600	-0.53280500	-0.84192700
O	-2.38856800	1.28911400	-0.54435300
O	2.35420000	-1.29469500	0.47601800
O	1.01548200	1.14123200	1.16518200
I	-1.76506900	-0.26325700	0.14431100
I	1.62649100	0.17226100	-0.18278900

$(I_2O_4)_2$

O	2.98141100	1.03665200	-0.64520600
O	0.91011400	-0.64535000	-0.59453100
O	3.48659200	-0.77754400	1.44646000
O	5.66404400	0.50949800	0.08133600
I	1.12517600	1.11052800	0.13370900
I	4.16633100	-0.39693500	-0.14624800
O	-2.98151000	-1.03678600	0.64501100
O	-0.91000400	0.64512400	0.59488100
O	-5.66390300	-0.50961600	-0.08252100
O	-3.48600400	0.77859200	-1.44582200
I	-1.12519000	-1.11056800	-0.13373400
I	-4.16643000	0.39688900	0.14633200

$(I_2O_4)_3$

I	-5.26159600	-0.62463500	-0.53638600
O	-3.52591100	-0.02074100	-1.06018400
I	-2.23613200	0.74437400	0.32795500
O	-1.46435000	-0.94243600	0.57337800
O	-4.89018800	-1.31647900	1.05135800
O	-6.07128900	0.92508100	-0.26222500
O	0.56747200	0.13778000	-0.90267000
I	0.36607900	-1.63639400	-0.39308200
O	2.24247100	-2.07854100	-1.08057600
I	3.78785200	-1.59575700	-0.06894800
O	4.16228900	-3.14353700	0.69204700
O	3.03937100	-0.63081000	1.24520000
O	1.83385800	2.17378800	0.71333300
O	-0.79615900	1.57728400	1.75712600
O	3.45903100	0.79673200	-1.31207200
O	2.97189100	3.54899600	-1.34056100
I	0.96447400	0.96284700	1.91276000
I	2.14860800	1.99452700	-1.25349100

Supporting Information

$(HIO_3)_1(I_2O_4)_1$

O	2.03130300	1.08683500	0.48032400
O	-0.27004300	-0.00156600	1.46506400
O	3.75991900	-0.08968800	-1.45409300
O	1.81421500	-1.64825600	-0.05286600
I	0.05223500	1.08550900	-0.06370900
I	3.11046400	-0.45116700	0.15039900
I	-3.10094700	-0.40596400	-0.28970500
O	-1.79583100	-1.62160800	-0.17390400
H	-0.49299000	-0.89052800	1.11320100
O	-2.02710400	1.14686200	-0.35794100
O	-3.85994900	-0.27426000	1.29923700

$(HIO_3)_1(I_2O_4)_2$

O	1.57770700	2.04942900	0.91975900
O	-0.07859400	-0.19541900	1.36535300
O	1.41091500	0.62425700	-1.52094200
O	2.70215100	3.09658600	-1.43533600
I	-0.43716400	1.59641000	1.08308800
I	2.62642400	1.56430500	-0.56753900
O	0.92885000	-2.13896000	-0.78134200
O	-1.71699100	-2.34411400	-1.56997600
O	2.56168600	-3.50739400	0.94003500
O	2.82785800	-0.72753900	0.80047400
I	-0.37962700	-0.98479300	-1.58965300
I	1.63137100	-2.01830100	1.06570200
I	-3.63352000	0.29292500	0.15605000
O	-3.23628100	-1.42660600	0.44863500
H	-2.31283000	-2.16181400	-0.80092700
O	-2.84743100	0.64926700	-1.40822800
O	-2.56534500	1.20584500	1.36351500

$(HIO_3)_1(I_2O_4)_3$

I	3.24460600	-0.72554000	-1.22151300
O	4.58811000	-1.46834600	-2.39224800
H	5.10803200	-2.13862600	-1.92851900
O	4.19527600	-0.85195900	0.26164900
O	2.28796000	-2.30486800	-1.24394700
O	2.39601000	1.48158100	2.13158200
O	1.54982700	0.00046500	0.00029700
O	0.74235100	2.99351000	0.40103300
O	3.33674400	2.17101500	-0.51080800
I	1.49522800	-0.41214800	1.83636000
I	2.45708700	2.86135000	0.87283400

O	-1.57525300	-3.05933900	0.24547200
O	0.93649700	-2.32128900	1.25832600
O	-1.58541200	-0.43035500	1.07207000
O	-3.48854900	-2.24399500	2.14294600
I	0.22618700	-2.68185500	-0.45882400
I	-2.86558600	-1.61175500	0.62400600
O	-2.54947900	2.28805700	-0.35113900
O	-4.39431700	0.25763500	0.70819100
O	-0.49670300	2.20478100	-2.38221700
O	-1.89988600	-0.08768900	-1.70052100
I	-4.31839300	1.57339700	-0.58381200
I	-0.94579800	1.24381500	-0.97842600

$(HIO_3)_2(I_2O_4)_1$

O	-2.31762500	-1.80339900	0.01729800
O	-0.40949300	-1.25081100	-1.85888700
O	-3.48984500	0.10888000	1.77586400
O	-2.14775300	0.95342900	-0.59249300
I	-0.20403300	-1.81839500	-0.05884800
I	-3.33360700	-0.22806000	0.05049200
I	0.15546500	2.37640200	-0.23265500
O	0.79586600	0.97493200	-1.17817600
H	-0.12967200	-0.29970600	-1.83679600
O	1.59105900	3.39284400	-0.07158200
O	0.02884000	1.58697900	1.46546200
I	3.00578800	-0.46442200	0.04588000
O	2.17511200	0.13429900	1.51660200
H	0.87498700	1.05381100	1.60455100
O	1.80997700	-1.88468800	-0.38746200
O	4.36426700	-1.41583400	0.63515000

$(HIO_3)_2(I_2O_4)_2$

I	-2.28537500	1.48321100	-0.88706700
O	-2.89121300	3.24922500	-0.37720100
H	-2.68706900	3.41655600	0.55161200
O	-1.89281900	1.04696500	0.80726000
O	-0.53051900	2.14932800	-1.28654400
O	-3.13936100	-1.76836400	0.70995400
O	-1.70198500	-0.50799200	-1.42770600
O	-5.81862000	-1.16871800	1.24247400
O	-4.27268500	0.60205100	-0.31218000
I	-1.17146900	-1.88011800	-0.26269000
I	-4.24005000	-0.38752500	1.23656800
I	2.28537800	-1.48317300	-0.88712200

O	2.89121500	-3.24919700	-0.37728300
H	2.68707000	-3.41653400	0.55152800
O	1.89284200	-1.04693800	0.80721000
O	0.53051800	-2.14928100	-1.28657400
O	3.13933800	1.76830900	0.71005900
O	1.70198800	0.50804300	-1.42773800
O	5.81859500	1.16862800	1.24260600
O	4.27269200	-0.60203400	-0.31221000
I	1.17149500	1.88016200	-0.26269600
I	4.24002400	0.38743800	1.23659000

$(HIO_3)_2(I_2O_4)_3$

O	0.01929700	-2.30373300	-2.30590100
O	-1.81123800	-3.40453100	-0.56261100
O	1.83889500	-0.47973300	-1.31732400
O	0.92046000	-2.59648300	0.26005100
I	-1.72141700	-1.74526800	-1.49040600
I	1.69520500	-2.26579500	-1.32711500
I	0.01894600	-0.79446300	1.83356600
O	-1.53167500	-1.69690300	1.72625800
H	-1.55415000	-3.19164100	0.35783500
O	1.03263600	-1.56592700	3.05744600
O	-0.57548100	0.64908900	2.96028400
I	-4.35773200	-0.46405800	0.73811800
O	-4.53602600	1.11778000	-0.23214200
H	-1.33355200	1.08269000	2.52672100
O	-3.72870800	-1.49102500	-0.64108200
O	-6.03262800	-0.98431700	0.84977700
O	-1.12487000	3.37041700	-0.63551800
O	1.58910800	2.83418500	-1.23145300
O	-2.23465700	1.53745300	0.96906100
O	-2.19728900	1.07170000	-1.81601500
I	0.43948800	2.59381100	0.27850300
I	-2.76102100	2.24946800	-0.61086100
O	4.61367400	0.34144000	0.09822800
O	3.62168400	-2.34247300	-0.29374600
O	4.40064200	3.12062700	-0.47063700
O	2.39184000	1.61325000	0.82956800
I	3.91961600	-1.11501000	1.09026200
I	3.32620600	1.76362500	-0.75430200

$(HIO_3)_3(I_2O_4)_1$

O	-2.42435100	-1.40394200	-1.51757700
O	-0.78603000	0.52132000	-2.87726000

O	-2.48246400	-1.31282300	1.32748500
O	-2.72054300	1.06654500	-0.18442600
I	-0.27457600	-0.87940700	-1.68872200
I	-3.31327100	-0.62735200	-0.09389600
I	-0.64725400	2.29636900	0.84012600
O	0.11106300	2.01598900	-0.76673600
H	-0.74425000	1.33887400	-2.33238300
O	-1.47461400	3.85256000	0.69015500
O	0.95779500	2.95308500	1.69451700
I	2.74635200	0.53228700	-0.60286800
O	3.10119000	-0.94327800	0.42132100
H	0.95651300	3.92061500	1.59249000
O	1.68974100	-0.33524900	-1.91645300
O	4.22362800	0.73306500	-1.53768600
I	1.27764400	-1.73550500	1.59525900
O	-0.19787800	-2.26386900	2.68101900
H	-1.04677300	-1.99510200	2.26249200
O	0.76139300	-0.02029900	1.38702300
O	0.74396300	-2.53099900	0.07771200

$(HIO_3)_3(I_2O_4)_2$

I	-1.88228200	-2.51123200	0.09129200
O	-2.02940000	-3.69825600	-1.43907400
H	-1.91030900	-3.19199800	-2.25234200
O	-1.35558600	-1.14455400	-0.93768400
O	-0.11235600	-3.17813100	0.46224100
O	-3.44283500	0.79657900	0.77838800
O	-1.70208200	-1.16356500	1.75129100
O	-5.62859000	0.52297300	-0.93549700
O	-3.91854000	-1.65547700	-0.40477600
I	-1.45892900	0.69080600	1.56493100
I	-3.91564400	0.12067800	-0.88476400
I	2.08890200	0.59665600	1.60155500
O	2.20033700	2.51307800	1.72202900
H	1.34163600	2.93151700	1.51979800
O	1.49421100	0.65023000	-0.11403700
O	0.40234100	0.57942900	2.43693100
O	3.53408900	-1.68286500	-1.04481600
O	1.96395500	-1.52007600	1.31915300
O	5.88565600	-0.22397200	-1.52010100
O	4.11857000	0.56874000	0.54208200
I	1.58910900	-2.43790900	-0.27420600
I	4.17159700	0.03759100	-1.20873000
I	-0.59925000	3.51003100	-0.94566300

O	0.90297400	2.58251800	-1.53836600
H	1.14727700	1.78791200	-0.88712400
O	-0.38515700	3.38477000	0.82965900
O	-1.95687300	2.43378900	-1.33670800

$(HIO_3)_3(I_2O_4)_3$

I	-2.47642800	-2.30552600	0.51287400
O	-3.50426800	-3.42314600	-0.73893300
H	-2.90232400	-3.86803100	-1.34730600
O	-2.39094000	-0.96807200	-0.68177300
O	-0.90099200	-3.17698900	-0.12823400
O	-4.75809400	0.96628400	-0.52073000
O	-1.49053600	-1.09109100	1.91670500
O	-7.03521300	-0.60470600	-0.10269100
O	-4.54423800	-1.49240100	0.88081800
I	-0.51599400	0.52229300	1.74042000
I	-5.35769600	-0.78814000	-0.59796900
I	2.68147600	-0.81919500	1.80214800
O	3.71454900	0.51845300	2.61763100
H	3.85947500	1.31007800	2.00214100
O	2.22142100	0.19657000	0.37140300
O	1.05229100	-0.27396300	2.68095800
O	2.62661900	-1.61642400	-1.78602500
O	1.59880700	-2.40382900	0.75903200
O	4.60577400	-3.52535800	-1.26735900
O	4.36313900	-1.40028600	0.51991700
I	0.79734400	-2.38847600	-0.92889800
I	4.39917500	-1.77327100	-1.28340700
I	3.16622600	1.99336700	-0.66487800
O	3.75135200	3.61259000	-1.53404900
H	4.63399900	3.85464800	-1.22151000
O	4.03459300	2.36304300	0.85483800
O	4.27448200	0.86919400	-1.51038400
O	-0.95261600	2.53895500	-1.21084400
O	0.87542100	2.86264800	0.98659700
O	-3.53140700	3.40788500	-0.80988900
O	-2.39111000	1.52924000	0.91482900
I	-0.22074000	3.90543600	-0.06900600
I	-2.82324100	1.79680400	-0.83445100

$(HIO_2)_1(I_2O_4)_1$

O	-1.79637000	1.03865800	-0.53554100
O	0.44913300	-0.39786100	-0.69078500
O	-4.29789500	-0.08536700	0.11296600

O	-1.91947300	-0.91673700	1.49591900
I	0.12158200	1.32462300	0.05247900
I	-2.63259900	-0.63473400	-0.10371100
O	2.22178100	1.10447400	0.33110400
I	2.56225400	-0.71728400	-0.14213000
O	4.43936700	-0.50755200	0.39791000
H	4.51209200	-0.43300200	1.35561300

$(HIO_2)_1(I_2O_4)_2$

O	-0.32610400	3.00744300	-0.00823900
O	0.91821900	1.16010700	1.55718800
O	-0.93326900	0.55582000	-0.98522200
O	-2.79142300	2.70061400	-1.37722800
I	1.45275300	2.16554700	0.03091300
I	-1.91080800	1.80621200	-0.14389400
H	0.64379100	0.22011100	1.26845300
O	0.29857800	-1.22292600	0.95185000
I	3.46611000	-0.75467700	0.32920600
O	3.39326000	1.12286900	0.28724700
O	-1.82596100	-2.28446700	-0.50555900
O	-3.50624600	0.15660400	-0.06664600
O	2.25323800	-1.19618600	-1.03992300
O	0.67218200	-3.55002000	-0.54190600
I	-2.95062700	-1.42742400	0.75063500
I	0.20929600	-1.86171400	-0.72989600

$(HIO_2)_1(I_2O_4)_3$

O	-1.91384800	0.62238800	2.17326600
O	-3.41544400	0.39592000	-0.26702700
O	-1.74604500	-2.05546900	1.53317900
O	0.26491300	-0.97469400	3.24444700
I	-3.71148100	0.34648200	1.56249900
I	-0.49698700	-0.79278300	1.66712000
O	3.69236500	1.27019000	0.30918400
O	3.46956000	-1.59116000	-0.18372400
O	2.49223100	3.87536500	0.01281100
O	0.97223800	1.61226900	0.83929800
I	3.49560600	-0.33210300	1.24027900
I	1.92365100	2.29996700	-0.52200600
O	-0.07610400	-2.72999500	-1.90728900
O	-1.82551600	-0.70667000	-3.09361400
O	0.98660900	-2.34548900	0.59572500
O	1.29300100	-0.39426900	-1.42179100
I	-1.73264800	-1.75996800	-1.59193200

I	1.63960100	-2.10752100	-1.07566700
H	-1.89349600	0.75632900	-2.87192400
O	-2.03222200	1.76208300	-2.66119600
I	-1.43245600	2.06540200	-0.90040700
O	0.15993300	3.02347200	-1.33101800

$(HIO_2)_2(I_2O_4)_1$

O	-1.87975900	-1.94624700	0.36160400
O	-2.62222900	0.95141400	0.48694700
O	0.13408100	-0.80268900	-1.10876500
O	0.12942700	-0.67623500	1.78408800
I	-2.98470400	-0.62002700	-0.43357200
I	0.23183100	-1.77368500	0.40530600
H	1.73872300	2.51360000	0.24879600
O	2.77720000	1.28159200	0.59718300
I	3.19833400	-0.16529300	-0.45120800
O	2.27007900	-1.66423000	0.33036400
H	-0.33262900	0.71328300	-1.47502500
O	-0.70363900	1.63636700	-1.48086700
I	-0.63935700	2.20337700	0.33941500
O	1.00363800	3.17270000	0.11061700

$(HIO_2)_2(I_2O_4)_2$

O	-3.86670400	-0.81359600	-0.16637800
O	-3.25095300	2.05776500	0.26492400
O	-2.08679100	-2.38974000	1.23655300
O	-1.21060700	-0.64007400	-0.80230900
I	-3.63048100	0.46813200	1.19125100
I	-2.17327900	-2.12962100	-0.51991700
O	3.86670900	0.81357700	-0.16635000
O	3.25096800	-2.05783000	0.26488200
O	2.08674800	2.38971100	1.23658700
O	1.21060500	0.64012800	-0.80232200
I	3.63049700	-0.46820800	1.19122900
I	2.17328700	2.12964900	-0.51988800
H	-1.36172100	0.72498000	-1.62239100
O	-1.49030700	1.68367000	-1.88177600
I	-1.18360800	2.67139300	-0.29754400
O	0.60212000	3.25490000	-0.92455500
H	1.36173600	-0.72499400	-1.62239200
O	1.49030900	-1.68367000	-1.88178800
I	1.18358500	-2.67134500	-0.29754600
O	-0.60210900	-3.25484400	-0.92462100

Supporting Information

$(HIO_2)_2(I_2O_4)_3$

O	3.19241500	-2.26795300	-0.52395000
O	3.80981000	0.44563600	-1.54569700
O	1.27723700	-0.58922800	0.33478000
O	2.95755200	-1.75940400	2.20582800
I	4.51943200	-0.93885600	-0.49759000
I	1.67988800	-2.25059000	1.08831100
O	-2.50657000	3.01806600	0.74258200
O	-3.00393000	0.49184000	2.06304900
O	-0.85744800	2.57641700	-1.43378300
O	-0.00855900	1.90509900	1.16082900
I	-3.28906600	1.32055300	0.39705400
I	-0.50258700	3.31069400	0.15160800
H	2.26874100	1.09657200	1.15831000
O	2.98280700	1.67359400	0.82524000
I	2.49777100	2.13226900	-0.96199600
O	1.41330700	3.64104100	-0.34229200
H	-0.28064500	1.09846200	2.63501100
O	-0.52660500	0.37388500	3.25909200
I	-1.38347000	-0.93463400	2.17797000
O	0.23136900	-2.13157400	2.35864900
O	-1.87245500	-0.98386800	-2.44644900
O	-0.51075200	-2.34877500	-0.57536700
O	-4.51243900	-1.81518000	-2.70428100
O	-3.45276300	-1.37094200	-0.11861700
I	-0.23160400	-0.62109900	-1.23201200
I	-3.11851400	-2.18936100	-1.68693300

$(HIO_2)_3(I_2O_4)_1$

O	-1.74830800	-2.33231800	-0.45075200
O	-3.70652500	-0.12557900	-0.31359000
O	0.05295500	-0.77368200	0.99764700
O	0.95193400	-3.05626500	-0.25963600
I	-3.08060600	-1.59725300	0.65742600
I	0.19168600	-1.49735100	-0.64296400
H	2.89302500	-3.02668600	-0.05806600
O	3.74226300	-2.55665300	-0.15191700
I	3.43963400	-0.67480400	0.27046800
O	2.02231000	-0.56632600	-0.99874600
H	0.20972600	0.71458700	1.33067300
O	0.42489200	1.68746900	1.54152700
I	1.43315800	2.33851000	0.06141500
O	3.07054700	1.52846800	0.46307000
O	-1.09816900	0.73856500	-1.15797000

I	-2.40108800	1.72237900	-0.30272900
O	-1.18951900	3.35956500	-0.20719100
H	-1.16935000	3.63763500	0.71627000

$(HIO_2)_3(I_2O_4)_2$

O	-2.44146700	-1.13205100	1.02791500
O	-0.61397900	-3.00869000	0.06866700
O	-0.52141400	0.16724500	-0.42156300
O	-2.10939200	1.63880700	1.35853900
I	-2.38255300	-2.32106600	-0.42416400
I	-0.84454100	0.36029700	1.34534400
H	2.49411300	3.38729200	0.05177800
O	2.95372500	2.97766200	0.88589400
I	2.46897700	1.15791300	0.92737100
O	0.61198900	1.60429200	1.62634800
H	-0.53117500	1.45158100	-1.43414100
O	-0.51758900	2.23480200	-2.05054700
I	-0.09684900	3.80342400	-1.03989700
O	1.74528100	3.88015200	-1.11866500
O	3.00972300	-1.42287500	-1.32586300
O	4.50985400	0.56079300	0.14440900
O	1.39666600	-1.31888400	1.00491800
O	2.00194300	-3.78787100	-0.20234000
I	4.58018500	-1.26324900	-0.30066800
I	1.16132800	-2.26917000	-0.52032700
O	-4.43004600	-1.40793900	-0.86677300
I	-5.11371000	0.04335000	0.07106200
O	-3.93721700	1.45718600	-0.38649100
H	-3.18791400	1.51059300	0.31462800

$(HIO_2)_3(I_2O_4)_3$

O	-0.57886600	3.70896600	-0.60127200
O	2.23252200	3.99574800	0.29566700
O	-1.12321500	1.17524600	0.39636400
O	0.52742500	1.37241100	-1.77435400
I	0.45800000	3.98070000	0.92751500
I	-1.18457800	1.68760400	-1.33286600
O	3.11402000	-3.31586300	-0.26925700
O	0.32327900	-3.66815100	-1.23936300
O	3.13119700	-0.99110200	1.27953400
O	2.30613800	-0.90153300	-1.37672200
I	1.38085500	-3.83023600	0.28889100
I	3.74173600	-1.26904600	-0.39195500
H	1.90009400	2.01268100	-1.95851100

O	2.84481800	2.37438100	-1.89978900
I	3.36495400	2.15198900	-0.10104700
O	4.46513600	0.54862700	-0.52636000
H	1.17447800	-1.18806500	-2.83208900
O	0.33161900	-1.56081400	-3.15652100
I	-0.78063800	-1.70945100	-1.61397600
O	-1.73036500	-0.02738400	-2.06795400
O	-1.12494100	-0.32034200	2.77761600
I	0.49225400	-0.29751900	1.83247200
O	1.26825500	-1.53196800	3.12250500
H	2.20186200	-1.60139600	2.85930400
O	-4.82322400	-0.51212900	0.72642300
O	-3.72999100	1.58573500	-0.97677900
O	-2.25789200	-1.61346200	0.52842400
O	-3.64036000	-1.67462100	2.99654800
I	-4.78986700	0.07232900	-1.08865800
I	-3.01405200	-0.56699700	1.78252200

$(HIO_3)_1(HIO_2)_1(I_2O_4)_1$

O	1.93981700	-1.72254700	0.23203600
O	-0.19450200	-1.00994300	1.84445700
O	4.35399300	-0.97246900	-0.95987600
O	2.11027500	0.72570900	-1.19679500
I	-0.11820100	-1.72223400	0.09826600
I	3.07488000	-0.22413900	-0.00649400
I	-3.30083200	-0.30487900	-0.23022000
O	-2.72188900	0.89528100	0.95489300
H	0.18982500	-0.05010100	1.73387400
O	-2.22814400	-1.79561000	0.15098300
O	-2.64686500	0.21674800	-1.79219800
H	-1.81029800	2.46563200	0.95071800
O	-1.06463100	3.08841800	0.83796500
I	0.29528700	2.12216200	-0.12092600
O	0.97824600	1.12769200	1.31132000

$(HIO_3)_1(HIO_2)_1(I_2O_4)_2$

O	1.51397000	-1.09934000	-1.64778000
O	-0.64696100	-2.89528100	-1.34853100
O	3.88277100	-2.50953200	-1.32024300
O	3.69391600	0.17273600	-0.53160200
I	-0.50527300	-0.98726300	-1.50856100
I	2.86415200	-1.44998300	-0.35694500
I	-3.85711400	-0.17580800	-0.38619300
O	-3.29260200	-0.89081100	1.14231700

H	-0.45590800	-3.06556500	-0.40388700
O	-2.76448300	-0.93401700	-1.63679500
O	-3.26654700	1.53092000	-0.33687500
H	-2.41352400	-1.88071100	2.53305400
O	-1.63138200	-2.06476200	3.08225200
I	-0.18942900	-1.05843800	2.28878000
O	0.46000900	-2.29470900	1.07611600
O	0.77607000	3.10733100	0.32933600
O	-0.48944600	1.47597400	-1.54146400
O	1.42470100	0.50522400	0.81435700
O	3.35589900	2.55589900	1.26522300
I	-1.01882600	2.38262300	-0.00482300
I	2.30539800	1.88640100	0.02623900

$(HIO_3)_1(HIO_2)_2(I_2O_4)_1$

O	3.29303100	1.16559600	0.37599100
O	1.47820600	1.64798700	-1.66658400
O	5.15807000	-0.61266300	1.45648200
O	2.51737300	-1.48820200	1.02144000
I	1.31193100	1.90832900	0.19952100
I	3.88872300	-0.60083700	0.23150100
I	-2.10318700	1.65710000	-0.25282100
O	-1.44585100	0.48340200	-1.42307200
H	1.44298200	0.63225800	-1.76793800
O	-0.52902700	2.73365500	-0.10819900
O	-1.89164800	0.85045400	1.33742700
H	-1.24310200	-1.27178400	-1.77540900
O	-0.83740200	-2.15598300	-1.68703800
I	0.62978300	-1.90886100	-0.43411900
O	1.71973100	-0.87278800	-1.54149200
O	-4.07539200	0.45895300	-0.36934400
I	-3.91222800	-1.24826700	0.34081700
O	-3.80843500	-0.82657100	2.20419000
H	-3.02535000	-0.22679700	2.24531800

$(HIO_3)_2(HIO_2)_1(I_2O_4)_1$

O	0.07972300	2.85377500	0.10148700
O	1.89121600	1.48901200	1.66855800
O	-2.60804300	3.24101300	-0.27500300
O	-1.35332700	0.90434300	-1.24089100
I	1.78950100	1.95733500	-0.17592800
I	-1.64843200	1.87027800	0.26249100
I	0.19899400	-2.00948100	0.16914800
O	-0.50826400	-1.42124800	-1.44536500

H	-0.83742900	-0.42214400	-1.34356300
O	-1.21711200	-2.91043600	0.79319500
O	0.12147700	-0.46476200	1.07673800
H	-2.69630000	-2.48690200	0.94644900
O	-3.66975600	-2.20569500	0.97493000
I	-3.93485100	-0.89898400	-0.37418200
O	-3.52994300	0.67424300	0.52760200
I	3.84204000	-0.93018200	-0.20785100
O	3.14336000	-1.35406900	1.37834100
H	1.48673800	0.59765000	1.74121700
O	3.78615500	0.90991300	-0.26519100
O	2.48234600	-1.35405900	-1.30052800

$(HIO_3)_2(HIO_2)_1(I_2O_4)_2$

O	1.69321000	-1.64934400	-1.66112300
O	-0.86856600	-2.44177400	-1.21695600
O	3.34244200	-3.86591900	-1.19500400
O	4.13074700	-1.28834800	-0.36936300
I	-0.07441100	-0.69230000	-1.50750500
I	2.76561100	-2.46044700	-0.30755000
I	-2.81184700	1.63313300	-0.63777400
O	-2.27221000	1.56015200	1.05233900
H	-0.73144000	-1.87377900	1.35363600
O	-2.10608200	0.18347300	-1.51107200
O	-1.46556600	2.83622300	-1.22636600
H	-2.06765600	-0.04550500	2.88470000
O	-1.29222500	-0.06945300	3.47299100
I	0.25031400	0.06869700	2.33001000
O	0.17089900	-1.75357800	1.73579600
O	2.46003000	2.68352300	0.28862800
O	0.99014800	1.48618700	-1.69382700
O	2.06350000	0.00180300	0.85555800
O	4.53927300	1.26023900	1.43373200
I	0.48778700	2.81313800	-0.47767400
I	3.38669700	1.05440600	0.12243100
I	-4.04899300	-1.60706500	0.40894800
O	-3.59662900	-2.44809100	-1.09727300
H	-1.84867100	-2.42300000	-1.33032000
O	-4.40275700	0.12710100	-0.09325700
O	-2.50816600	-1.44275500	1.31957700

$(HIO_3)_2(HIO_2)_2(I_2O_4)_1$

O	-0.54595900	2.82128900	0.47281200
O	-2.35100700	1.96929000	-1.40432300

O	1.19052800	1.03855200	1.56680200
O	0.53246400	0.88958400	-1.19711700
I	-2.38199200	1.78019900	0.48208500
I	1.10875000	2.04082300	0.05234100
I	-4.50374800	-0.94474900	-0.15709800
O	-3.30555800	-1.73786900	0.91021600
H	-2.12113600	1.04291900	-1.71463100
O	-4.20360400	0.88160200	0.30644000
O	-6.06418700	-1.15211000	0.64020600
H	-2.14884300	-3.00610300	0.13183000
O	-1.47070000	-3.16181500	-0.55612500
I	-0.64528100	-1.43679900	-0.81184000
O	-2.10687400	-0.58877000	-1.58850100
O	2.22311600	-1.17511100	0.20147200
I	2.64180700	-0.76427200	1.99534200
O	3.82010400	-2.29047600	2.18072200
H	4.55625800	-2.13455900	1.56491900
I	3.58186400	-0.34653900	-1.25268300
O	4.75083800	0.43983600	-2.57610800
H	5.56037400	0.77566500	-2.16894800
O	3.20932100	1.27156200	-0.48580900
O	4.90640900	-0.96769200	-0.23880500

$(HIO_3)_3(HIO_2)_1(I_2O_4)_1$

O	-2.17462500	1.12744400	1.41870900
O	-2.63894600	3.28396100	-0.34317800
O	-0.29971400	0.52105200	3.30788000
O	-1.37417100	-1.49577300	1.73732300
I	-2.94656200	1.40621500	-0.30343900
I	-0.43384000	0.09949400	1.61936300
I	1.06980300	2.63520400	-0.63446500
O	-0.18133200	3.82256000	-1.11644000
H	-1.70555800	3.49942900	-0.64474300
O	0.38031200	1.14334100	-1.43457200
O	0.65014500	2.32072000	1.08983200
I	3.25093900	-0.68388000	1.33339400
O	3.13787500	0.74204500	0.23352000
H	3.31484800	0.32966900	-1.59947500
O	3.65270400	-2.01516800	0.21991300
O	1.50650500	-1.04729200	1.75035500
H	2.82911600	-2.79027700	-1.44706200
O	2.26925800	-2.64047500	-2.23021600
I	1.35996000	-0.97035900	-1.91399800
O	2.86654400	0.09637500	-2.43622800

I	-2.35300700	-2.39639100	0.04643000
O	-3.22097200	-3.35986800	-1.39279900
H	-3.15768400	-2.89481300	-2.23632200
O	-3.43692900	-0.95834800	-0.09092400
O	-0.94756100	-1.90670700	-0.94797800

$(HIO_3)_3(HIO_2)_1(I_2O_4)_2$

O	-1.36816000	-2.46104300	-0.45167100
O	-0.95285900	-3.40947800	2.18178200
O	0.27877000	-1.39010300	-2.26327600
O	-2.32456300	-0.49785900	-2.14327300
I	-2.35191700	-2.62502200	1.16921200
I	-0.81635900	-0.59196800	-1.10627200
I	1.02817400	-0.19806400	2.12508000
O	0.74531500	-1.67179600	3.09709600
H	-0.30812900	-2.71522700	2.54139700
O	-0.73417500	0.32999200	1.95463100
O	1.19129300	-0.91621000	0.45905300
I	1.19374200	2.45048900	-1.36601400
O	1.57985800	2.02538200	0.34265600
H	0.76486100	2.98676400	1.67997300
O	0.22488500	3.92634800	-1.13097900
O	-0.19552400	1.29570100	-1.82112100
H	-1.39375400	4.42371300	-0.01358400
O	-1.97738300	4.23090100	0.74277200
I	-1.50851000	2.44633200	1.32253700
O	0.01780900	3.05828400	2.30953400
I	-4.26769300	-0.12005000	-1.14864900
O	-6.00472100	0.33338700	-0.44044600
H	-5.99233800	0.42144900	0.52140700
O	-4.14904900	-1.70424500	-0.30456500
O	-3.35955000	0.96087300	-0.04967900
O	4.34551600	-1.43152900	-0.54728000
O	3.52842500	-0.62086800	2.17564300
O	3.49948700	-1.94509600	-3.22037300
O	2.64335300	0.32366400	-1.84309200
I	4.59512400	-0.06370900	0.78492200
I	2.71271400	-1.46040600	-1.72626000

$(HIO_3)_3(HIO_2)_2(I_2O_4)_1$

O	-0.74900000	-0.93665200	-2.75880400
O	-1.89250300	1.69234300	-2.57824500
O	0.87211700	-3.12896500	-2.22243600
O	2.02592000	-0.74950500	-2.91539100

I	-2.26659100	0.02634200	-1.74431600
I	0.83275400	-1.27958700	-1.74577600
I	-2.23707500	-2.56775600	0.92132000
O	-0.54512700	-2.88027700	0.38239500
H	0.39249200	-3.61199500	-1.52922100
O	-3.09440100	-2.20883700	-0.61236100
O	-2.02982600	-0.92842800	1.68044500
I	4.09539700	-0.19905100	-0.11089100
O	3.33218800	1.37486600	-0.52563700
H	0.47950500	1.67171100	2.28955200
O	2.73861100	-1.40513100	-0.14954900
O	4.34720300	-0.06932500	1.66341300
I	1.08132200	2.57810600	-0.53482000
O	-0.50704000	3.36764900	-0.93895000
H	-1.35160100	2.28018500	-1.99043500
O	0.51559100	0.88704000	-0.85339900
O	1.05452200	2.63484200	1.25887600
H	3.16812600	-0.43394700	2.57023400
O	2.42062000	-0.74693000	3.19199500
I	0.73554500	-0.70495100	2.33987500
O	0.12167400	0.98344600	2.95033200
O	-3.59077500	1.07829700	-0.34167000
I	-2.62808900	1.69890700	1.15376200
O	-2.57091100	3.55626800	0.73480000
H	-1.78234800	3.67191200	0.13821000
